# Management of Thyroid Eye Disease: A Consensus Statement by the American Thyroid Association and the European Thyroid Association

**DOI:** 10.1089/thy.2022.0251

**Published:** 2022-12-13

**Authors:** Henry B. Burch, Petros Perros, Tomasz Bednarczuk, David S. Cooper, Peter J. Dolman, Angela M. Leung, Ilse Mombaerts, Mario Salvi, Marius N. Stan

**Affiliations:** ^1^ National Institute of Diabetes and Digestive and Kidney Diseases, National Institutes of Health, Bethesda, Maryland, USA.; ^2^ Department of Medicine, Uniformed Services University of the Health Sciences, Bethesda, Maryland, USA.; ^3^ Endocrinology Division, Department of Medicine, Walter Reed National Military Medical Center, Bethesda, Maryland, USA.; ^4^ Department of Endocrinology, Leazes Wing, Royal Victoria Infirmary, Newcastle upon Tyne, United Kingdom.; ^5^ Department of Internal Medicine and Endocrinology, Medical University of Warsaw, Warsaw, Poland.; ^6^ Department of Medicine, Division of Endocrinology, Diabetes, and Metabolism, The Johns Hopkins University School of Medicine, Baltimore, Maryland, USA.; ^7^ Department of Ophthalmology and Visual Sciences, University of British Columbia, Vancouver, Canada.; ^8^ Division of Endocrinology, Diabetes, and Metabolism, Department of Medicine, UCLA David Geffen School of Medicine, VA Greater Los Angeles Healthcare System, Los Angeles, California, USA.; ^9^ Department of Ophthalmology, University Hospitals Leuven, Leuven, Belgium.; ^10^ Department of Clinical and Community Services, Graves' Orbitopathy Center, Endocrinology, Fondazione IRCCS Cà Granda, Milan, Italy.; ^11^ Division of Endocrinology, Diabetes and Metabolism, Mayo Clinic, Rochester, Minnesota, USA.

**Keywords:** thyroid eye disease, consensus statement, American Thyroid Association, European Thyroid Association

## Abstract

Thyroid eye disease (TED) remains challenging for clinicians to evaluate and manage. Novel therapies have recently emerged, and their specific roles are still being determined. Most patients with TED develop eye manifestations while being treated for hyperthyroidism and under the care of endocrinologists. Endocrinologists, therefore, have a key role in diagnosis, initial management, and selection of patients who require referral to specialist care. Given that the need for guidance to endocrinologists charged with meeting the needs of patients with TED transcends national borders, and to maximize an international exchange of knowledge and practices, the American Thyroid Association and European Thyroid Association joined forces to produce this consensus statement.

1. Summary of Key Points    1440
2. Introduction    1441
  2.1 Methods    1442
3. Background    1442
  3.1. Epidemiology    1442
  3.2. Natural history    1443
  3.3. Pathogenesis    1443
  3.4. Risks for TED development and opportunities for prevention    1443
  3.5. Early diagnosis and referral for TED specialty care    1443
  3.6. Role of endocrinologists and ophthalmologists in the care of patients with TED    1444
4. Patient Assessment    1445
  4.1. Assessing disease activity and severity    1445
  4.2. Assessment of quality of life    1446
  4.3. Formal ophthalmology evaluation    1446
  4.4. Imaging    1448
5. Overall Approach to Therapy    1449
  5.1. Local and lifestyle measures    1449
  5.2. Overview of systemic medical and surgical treatments for TED    1449
  5.3. Setting for TED care    1449
  5.4. Referral to ophthalmology    1449
6. Therapy for Mild TED    1451
  6.1. Medical therapy for mild TED    1451
  6.2. Surgery for minimal changes in proptosis and lid retraction    1451
7. Management of Moderate-to-Severe TED    1451
  7.1. Medical therapies    1451
    7.1.1. Glucocorticoids    1453
    7.1.2. Therapies for patients with moderate-to-severe TED unresponsive or intolerant to intravenous glucocorticoids    1456
    7.1.3. Teprotumumab    1457
    7.1.4. Rituximab    1458
    7.1.5. Mycophenolate    1459
    7.1.6. Tocilizumab    1460
    7.1.7. Other agents    1461
      7.1.7.1. Other agents tested in TED patients and clinically available    1461
      7.1.7.2. Other agents under investigation in TED patients but not clinically available    1461
      7.1.7.3. Other agents tested in GD patients with potential benefit in TED but not clinically available    1461
  7.2. Radiotherapy for moderate-to-severe TED    1461
  7.3. Surgical intervention for inactive moderate-to-severe TED    1462
    7.3.1. Surgical intervention overview    1462
    7.3.2. Orbital decompression    1462
    7.3.3. Strabismus procedures    1463
    7.3.4. Eyelid procedures    1463
8. Therapy for Sight-Threatening TED    1463
  8.1. Intravenous glucocorticoids    1463
  8.2. Radiotherapy in dysthyroid optic neuropathy    1464
  8.3. Orbital decompression for dysthyroid optic neuropathy    1464
9. Overview of the Management of TED    1465
10. Research Gaps in the Management of TED    1465

## 1. SUMMARY OF KEY POINTS

### 1.1. Diagnosis and assessment

**Key Point 3.1:** Early diagnosis of TED and simple measures to prevent TED development or progression should be pursued.

**Key Point 3.2:** Endocrinologists managing patients with Graves' disease should identify referral pathways that ensure patient access to TED specialty care.

**Key Point 3.3:** Ophthalmologists are key to the management of TED and should always be involved in the care of patients with moderate-to-severe and sight-threatening TED.

**Key Point 4.1.1:** Endocrinologists should be familiar with basic elements of a TED examination enabling assessment of both activity and severity.

**Key Point 4.1.2:** Assessment of patients with TED should include activity, severity (with particular attention to impaired ocular motility and visual loss), trend across time, and impact on daily living.

**Key Point 4.2.1:** The physical and psychosocial impact of TED should be assessed for each patient, as it informs treatment decisions. When formal quantification of quality of life (QOL) is deemed appropriate, Graves' orbitopathy-quality of life (GO-QOL) is the preferred instrument.

**Key Point 4.4.1:** Orbital imaging using contrast-enhanced computed tomography (CT) or magnetic resonance imaging (MRI) is preferred for atypical or severe cases of TED to help determine activity and to exclude other etiologies that could be confused with TED.

**Key Point 4.4.2:** Noncontrast CT is the preferred modality in patients with TED who are being considered for surgery.

### 1.2. Initial care and referral for specialty care

**Key Point 5.1.1:** Local ocular measures and lifestyle intervention should be offered to all patients with TED. Lubricants and nocturnal eye masks may be used to prevent or treat corneal exposure. Ocular occlusion and prisms may be offered to relieve diplopia. The importance of smoking reduction or cessation should be explained, and smokers offered support for this goal.

**Key Point 5.3.1:** Input from both endocrinologists and ophthalmologists with TED expertise is recommended for optimal management in patients with moderate-to-severe and sight-threatening TED.

**Key Point 5.4.1:** An ophthalmologist should be consulted when the diagnosis of TED is uncertain, in cases of moderate-to-severe TED, and when surgical intervention needs to be considered. Urgent referral is required when sight-threatening TED is suspected or confirmed.

**Key Point 6.1.1:** A single course of selenium selenite 100 μg twice daily for 6 months may be considered for patients with mild, active TED, particularly in regions of selenium insufficiency.

**Key Point 6.2.1:** The clinician should regularly assess the psychosocial impact of concerns about appearance.

### 1.3. Therapy of moderate–severe TED

**Key Point 7.1.1:** Infusion therapies for TED should be administered in a facility with appropriate monitoring under the supervision of experienced staff. Awareness and surveillance for adverse side effects are recommended throughout the treatment period.

**Key Point 7.1.2:** Clinicians should balance the demonstrated efficacy of recently introduced therapies against the absence of experience on sustained long-term efficacy, safety, and cost-effectiveness.

**Key Point 7.1.1.1:** Intravenous glucocorticoid (IVGC) therapy is a preferred treatment for active moderate-to-severe TED when disease activity is the prominent feature in the absence of either significant proptosis (see Section 2.1. for definition) or diplopia.

**Key Point 7.1.1.2:** Standard dosing with IVGC consists of intravenous methylprednisolone (IVMP) at cumulative doses of 4.5 g over ∼3 months (0.5 g weekly × 6 weeks followed by 0.25 g weekly for an additional 6 weeks).

**Key Point 7.1.1.3:** Poor response to IVMP at 6 weeks should prompt consideration for treatment withdrawal and evaluation of other therapies. Clinicians should be alert for worsening diplopia or onset of dysthyroid optic neuropathy (DON) that have occurred even while on IVMP therapy.

**Key Point 7.1.1.4:** A cumulative dose of IVMP >8.0 g should be avoided.

**Key Point 7.1.2.1:** Rituximab (RTX) and tocilizumab (TCZ) may be considered for TED inactivation in glucocorticoid (GC)-resistant patients with active moderate-to-severe TED. Teprotumumab (TEP) has not been evaluated in this setting.

**Key Point 7.1.3.1** TEP is a preferred therapy, if available, in patients with active moderate-to-severe TED with significant proptosis (see Section 2.1. for definition) and/or diplopia.

**Key Point 7.1.4.1:** Evidence from randomized controlled trials (RCTs) is limited and divergent but suggests efficacy of RTX for inactivation of TED and prevention of relapses at >1 year, particularly in patients with TED of <9 months' duration.

**Key Point 7.1.4.2:** RTX therapy is acceptable in patients with active moderate-to-severe TED and prominent soft tissue involvement.

**Key Point 7.1.6.1:** TCZ is an acceptable treatment for TED inactivation in GC-resistant patients with active moderate-to-severe disease.

**Key Point 7.2.1:** Radiotherapy (RT) is a preferred treatment in patients with active moderate-to-severe TED whose principal feature is progressive diplopia.

**Key Point 7.2.2:** RT should be used cautiously in diabetic patients to avoid possible retinopathy. It is relatively contraindicated for those younger than 35 years of age to avoid a theoretical lifetime risk of tumors developing in the radiation field.

**Key Point 7.3.1.1:** Surgery for moderate-to-severe TED should be performed by an orbital surgeon experienced with these procedures and their complications.

**Key Point 7.3.1.2:** Rehabilitative surgery for moderate-to-severe TED should only be performed when the disease is inactive and euthyroidism has been achieved and maintained.

**Key Point 7.3.2.1:** The specific surgical approach should be tailored to the indication (DON, proptosis), type of orbitopathy (muscle or fat predominant congestive disease), and desired reduction in proptosis.

**Key Point 7.3.3.2:** In patients with diplopia and inactive TED, binocular single vision in the primary position of gaze may be restored with strabismus surgery or permanent prisms ground into the spectacle lenses.

**Key Point 7.3.4.1**: Eyelid retraction and fat prolapse are surgically corrected when TED is inactive and euthyroidism is achieved, and after surgical decompression and strabismus surgery as indicated.

### 1.4. Therapy of sight-threatening TED

**Key Point 8.1.1:** Patients with DON require urgent treatment with IVGC therapy, with close monitoring of response and early (after two weeks) consideration for decompression surgery if baseline visual function is not restored and maintained with medical therapy.

**Key Point 8.2.1:** RT may be considered for preventing or as an adjunct to treating DON.

**Key Point 8.3.1:** In patients with compressive DON, orbital decompression of the deep medial wall and orbital floor should be considered to restore vision by reducing apical compression on the optic nerve.

## 2. INTRODUCTION

Thyroid eye disease (TED) is an autoimmune condition closely related to Graves' disease. It is characterized by endomysial interstitial edema, expansion, and proliferation of cells within the fibrofatty compartment, resulting in the clinical manifestations of periorbital edema, lid retraction, proptosis, diplopia, corneal breakdown, and in rare cases optic nerve compression. TED remains challenging for clinicians to evaluate and manage. Novel therapies have recently emerged, and their specific roles are still being determined.

Most patients with TED develop eye disease while being treated for hyperthyroidism under the care of endocrinologists. Endocrinologists, therefore, have a key role in diagnosis, initial management, and selection of patients who require referral to specialist care. Given that the need for guidance to endocrinologists charged with meeting the needs of patients with TED transcends national borders, and to maximize an international exchange of knowledge and practices, the American Thyroid Association (ATA) and European Thyroid Association (ETA) joined forces to produce this consensus statement (CS).

The scope was to address clinical assessment, to develop criteria for referral to specialty care and treatment, and to focus on medical and surgical treatment in nonpregnant adults (age ≥18 years) with TED. This CS is primarily aimed at endocrinologists and, in particular, those involved in the management of nonpregnant adult (>18 years) patients with TED. A CS was selected as the forum, rather than a clinical practice guideline, to provide a concise and timely appraisal of a rapidly changing therapeutic arena.

In line with the official policies of the ATA and ETA, this CS is intended as an aid to practicing endocrinologists. It does not establish a standard of care, replace sound clinical judgment, or capture all nuances likely to be present in any particular patient; specific outcomes are not guaranteed. We recommend that treatment decisions be based on independent judgments of health care providers carefully considering each patient's individual circumstances such as comorbidities, functional status, goals of care (established at the outset and revisited frequently), and feasibility considerations, including regional access to specific health care resources. Our recommendations are not intended to supplant patient directives.

A recent survey of ATA and ETA members^1^ found that 53% reported no access to a multidisciplinary clinic, and the cost of some medical treatments was deemed to be a barrier. The CS has taken this important information into account and has striven to achieve a balance between the limitations imposed by the above constraints and encouraging best practice.

### 2.1. Methods

Membership in the task force (TF) included physicians with expertise in thyroidology and TED, and adherence to the rules of the ATA and ETA on conflicts of interest (https://www.thyroid.org/wp-content/uploads/members/fin-disclosure-coi-policies-2018.pdf; https://www.eurothyroid.com/files/download/ETA-Rules-for-Guidelines-2016.pdf). Cochairs were nominated by ATA and ETA leadership and invited to suggest up to four additional individuals to represent the ATA and ETA. Potential members were discussed and vetted with ATA and ETA society leadership before the final taskforce was assembled.

A series of twice-monthly virtual meetings of the TF with an average attendance of 88% of members took place between January and November 2021, complemented by additional communications. A literature search of PubMed was initially conducted of English language publications from January 1990 through January 2021 and continuously updated up until the time of publication, using the search terms “thyroid eye disease” or “Graves' orbitopathy” or “Graves' ophthalmopathy” or “thyroid-associated eye disease.” References were imported into EndNote and the final database included 3952 unique references. The scope was discussed, agreed upon, and endorsed by the ATA and ETA. A detailed list of subtopics was constructed with approximate word and reference limits assigned to writing groups based on expertise.

Section drafts were reviewed by the TF. Recommendations were listed as “Key Points,” and discussed and modified until full consensus was reached. Specifically, for topics in which there were differing views among taskforce members, a comprehensive discussion took place, allowing iterative modification of the topic content until there was unanimous consensus. The final drafts were approved by the entire TF. Two patient-led organizations, the Graves' Disease and Thyroid Foundation and the Thyroid Organization of the Netherlands, were invited to review the final draft.

In addition, the CS was posted on the ATA and ETA websites for comments and feedback from members. Feedback was also received from the American Academy of Ophthalmology and the American Society of Ophthalmic Plastic and Reconstructive Surgery; the European Society of Ophthalmic Plastic and Reconstructive Surgery was invited to review the CS, but no feedback was received.

The TF chose the descriptor “TED” because it is commonly used in the literature and is meaningful to specialists, generalists, patients, and the general public, although the TF acknowledges that Graves' orbitopathy is also a widely accepted and frequently used term. Multidisciplinary specialized TED care, described hereunder (see Section 3.5), will be referred to as “TED specialty care.”

Several medical therapies are available for TED. Many have not been compared with placebo or compared with one another in randomized controlled studies. Therefore, the TF has categorized treatments as (1) preferable, (2) acceptable, or (3) may be considered, based on its collective interpretation of the available evidence. A treatment is listed as “preferred” if more than or equal to two RCTs have shown efficacy against standard of care or placebo with concordant results; “acceptable” when there exist more than or equal to two RCTs with discordant results but the discordance is deemed likely the result of differing inclusion criteria, or only a single RCT is available and shows efficacy.

Notably, most included RCTs were not placebo-controlled, but, rather, compared with other existing therapies. A therapy is listed as “may be considered” in the case of therapies for which benefit is not clear. Evidence for efficacy in this category may be the result of more than or equal to two RCTs with discordant results that are not easily explicable, or from single RCTs with small efficacy effects, and from larger well-performed observational studies. In general, therapies in the “may be considered” category are utilized in clinical practice only when both preferable and acceptable therapies are unavailable, contraindicated, or the patient is intolerant and/or refuses.

These definitions leave open the possibility of more than one preferable therapy for a given patient, in which case drug availability, cost, and patient acceptability are paramount in selecting the appropriate therapy for a particular patient. The TF is aware that regional differences currently exist in the availability of individual medical therapies and, therefore, some treatments listed as preferable will not be available in all regions of the world.

For therapies selected to reduce proptosis, the TF elected to use the term “significant proptosis” rather than a numerical threshold (i.e., ≥3 mm above the upper limit for race and sex) as a numerical definition would exclude some patients who might otherwise benefit from therapy. In keeping with the definition of moderate-to-severe TED (Table 1), a degree of proptosis <3 mm above the upper limit for race and sex would be regarded as “significant proptosis” if it impacted sufficiently on daily life and would justify the risks of treatment.

**Table 1.** Activity and Severity Definitions for Patients with Thyroid Eye Disease

A. Activity
1. Clinical activity score
The 7-item CAS is shown hereunder. Each item scores 1 point if present^a^
Spontaneous retrobulbar pain
Pain on attempted up or lateral gaze
Redness of the eyelids
Redness of the conjunctiva
Swelling of the eyelids
Inflammation of the caruncle and/or plica (Fig. 2b)
Conjunctival edema, also known as chemosis (Fig. 2c)
2. Active TED
A CAS ≥3/7 usually implies active TED. A history or documentation of progression of TED based on subjective or objective worsening of vision, soft tissue inflammation, motility, or proptosis is suggestive of active TED independently of the CAS
B. Severity
1. Sight-threatening TED
Patients with DON and/or corneal breakdown and/or globe subluxation (Fig. 2f)
2. Moderate-to-severe TED
Patients without sight-threatening disease whose eye disease has sufficient impact on daily life to justify the risks of medical or surgical intervention. Patients with moderate-to-severe TED usually have any one or more of the following: lid retraction ≥2 mm, moderate or severe soft tissue involvement, proptosis ≥3 mm above normal for race and sex, or diplopia (Gorman score 2–3).
3. Mild TED
Patients whose features of TED have only a minor impact on daily life insufficient to justify immunosuppressive or surgical treatment. They usually have only one or more of the following: minor lid retraction (<2 mm), mild soft tissue involvement, proptosis <3 mm above normal for race and sex, transient or no diplopia, and corneal exposure responsive to lubricants.

^a^ A 10-item CAS is also sometimes used and includes additional points for increase of at least 2 mm in proptosis, decrease of at least 8° in any duction, and decrease of visual acuity by two lines. A limitation of the 10-item CAS is that it requires an earlier assessment of the mentioned measures, which is usually unavailable on first consultation. See Bartalena *et al.*^19^

CAS, clinical activity score; DON, dysthyroid optic neuropathy; TED, thyroid eye disease.

## 3. BACKGROUND

### 3.1. Epidemiology

There is a close temporal relationship between the onset of hyperthyroidism due to Graves' disease (GD) and TED for patients in whom both disorders occur; in 80% of such cases, both hyperthyroidism and TED develop within 2 years.^2^ Rarely, TED occurs in euthyroid patients or in those with a history of chronic autoimmune thyroiditis. Notably, TED is almost always seen in conjunction with circulating thyrotropin (TSH) receptor antibodies (TRAbs).^3,4^

The overall prevalence of TED among patients with GD is up to 40%.^5^ Recent studies indicate that the clinical phenotype of GD at onset is becoming milder with respect to the prevalence and severity of hyperthyroidism, goiter, and TED.^6^ Moderate-to-severe and sight-threatening TED now occur in ∼6% and 0.5% of patients with GD, respectively.^7^ Moreover, TED is a heterogeneous disorder and some clinical variants of the disease (e.g., euthyroid TED) are considered rare.^8^

### 3.2. Natural history

The initial description of three phases of TED by Rundle and Wilson remains the widely accepted representation of its natural history.^9^ An initial active phase is characterized by inflammatory changes, followed by a brief static phase, and lastly by the inactive phase, which patients usually enter 12–18 months after disease onset. Although improvement in signs and symptoms occurs during the latter period, proptosis and extraocular muscle dysfunction frequently do not normalize without intervention and may persist in up to 50% of patients.^9^

### 3.3. Pathogenesis

TED develops from an autoimmune-mediated inflammation targeting connective tissue within and around extraocular muscles (EOMs), intraorbital fat, and less frequently lacrimal glands of some patients with GD.^2,10^ The close link between TED and TRAb supports the hypothesis that the TSH receptor (TSHR) is the primary autoantigen. The insulin-like growth factor-1 receptor (IGF-1R), with which TSHR forms a functional signaling complex on orbital fibroblasts, seems also to be involved in orbital inflammation, adipogenesis, and tissue remodeling.^11^

The histopathological changes correlate with the natural history and provide a mechanical basis for understanding the clinical features of TED. Infiltration of orbital tissues by lymphocytes and accumulation of hydrophilic glycosaminoglycans, interstitial edema, and increased adipogenesis are the characteristic findings in the active phase of disease. Increased fibrosis and fat infiltration of affected tissues are observed in the inactive phase.^2,10^

### 3.4. Risks for TED development and opportunities for prevention

Nonmodifiable risks for the development and severity of TED include older age, male sex, and genetic factors. The potential role of race in TED remains unclear,^7^ with anatomic differences in both normal and TED orbits postulated to account for variable presentation by race.^12^

Modifiable risk factors include cigarette smoking, thyroid dysfunction, and the use of radioactive iodine (RAI). Additional potentially modifiable factors are oxidative stress and elevated serum TRAb levels, the latter affected by choice of therapy for hyperthyroidism.^7^ Epidemiological studies have recently shown that statin therapy is associated with a decreased risk of developing TED in patients with GD.^13–15^

**FIG. 1.**
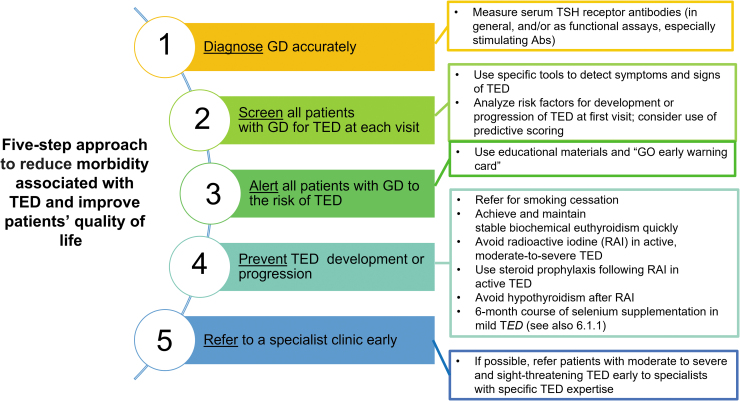
Steps to Reduce Morbidity and Improve Quality of Life in Patients with TED. Measures to reduce morbidity associated with TED and improve patients' QOL. (This figure is used and adapted with permission, courtesy of the British Thyroid Foundation, from the Thyroid Eye Disease Amsterdam Declaration Implementation Group UK (TEAMeD) (https://www.btf-thyroid.org/teamed-page) and Dr. Anna Mitchell. The Thyroid Eye Disease Amsterdam Declaration is further described in references 17, 20). Abs, antibodies; GD, Graves' disease; RAI, radioiodine; TED, thyroid eye disease.

The use of steroid prophylaxis in those receiving RAI and normalization of thyroid hormone levels and selenium supplementation in those with mild active disease may alter the natural history of TED^7^ (Fig. 1). Moreover, based on four independent variables (clinical activity score [CAS], serum TRAb levels, duration of hyperthyroidism, and smoking), a quantitative predictive score for identifying patients with GD least likely to develop TED (negative predictive value of 0.91) has been proposed.^16^ The low positive predictive value (0.28) of this predictive score limits the utility in predicting future TED.

### 3.5. Early diagnosis and referral for TED specialty care

Adoption of a set of simple measures to promote early diagnosis and prevention of TED is recommended by professional organizations,^17–19^ following the Amsterdam Declaration.^20^ It is important that endocrinologists have access to specialized clinical services for patients with TED. Five components are essential for optimal management of patients with TED:

- Multidisciplinary decision making based on close communication between experts and patients, utilizing shared decision making.
- Coordinated care that encompasses the management of both thyroid and orbital disease.
- Skills and expertise for the diagnosis, assessment, and treatment by specialists in TED from endocrinology, ophthalmology, orthoptics (for motility testing and prism fitting) and, as needed, otolaryngology/maxillofacial/plastic surgery, clinical psychology/counseling (with expertise in coping skills related to the impairment of QOL related to TED), nuclear medicine, radiology, and radiation oncology.
- Availability of evidence-based treatments.
- Safe and timely delivery of treatments.

The format of such a service may be a “Combined Thyroid Eye Clinic,”^21^ variants of this model in a physical or virtual setting, or a combination of both. The organizational details vary between countries and health care systems and are less important than satisfying the mentioned components. While a combined TED clinic structure can promote quality care in a timely manner,^22,23^ there is no clear evidence that this model of care is superior to others, and delivery of multidisciplinary care is more important than the structure of the clinic.

### 3.6. Role of endocrinologists and ophthalmologists in the care of patients with TED

Endocrinologists

- manage the thyroid dysfunction,
- diagnose TED among their patients with GD,
- initiate local and lifestyle measures (Section 5.1),
- consider checking selenium level (as indicated), 25-hydroxyvitamin D levels, and lipid levels (optional),
- refer to ophthalmologists those patients in whom the diagnosis or severity of TED is unclear, and all cases of moderate-to-severe and sight-threatening TED, and
- contribute to TED specialty care management decisions including the delivery of systemic therapies, and monitor for adverse events (AEs) of such therapies.

General ophthalmologists:

- Diagnose/confirm TED
- Provide emergency management of sight-threatening TED after hours
- Refer patients with moderate-to-severe or sight-threatening TED to specialty TED care

TED specialty care (Section 3.5)

- Diagnose/confirm TED
- Medical and surgical management of moderate-to-severe and sight-threatening TED
- Ensure optimal management of thyroid disease

**Key Point 3.1:** Early diagnosis of TED and simple measures to prevent TED development or progression should be pursued.

**Key Point 3.2:** Endocrinologists managing patients with GD should identify referral pathways that ensure patient access to TED specialty care.

**Key Point 3.3:** Ophthalmologists are key to the management of TED and should always be involved in the care of patients with moderate-to-severe and sight-threatening TED.

## 4. PATIENT ASSESSMENT

### 4.1. Assessing disease activity and severity

**FIG. 2.**
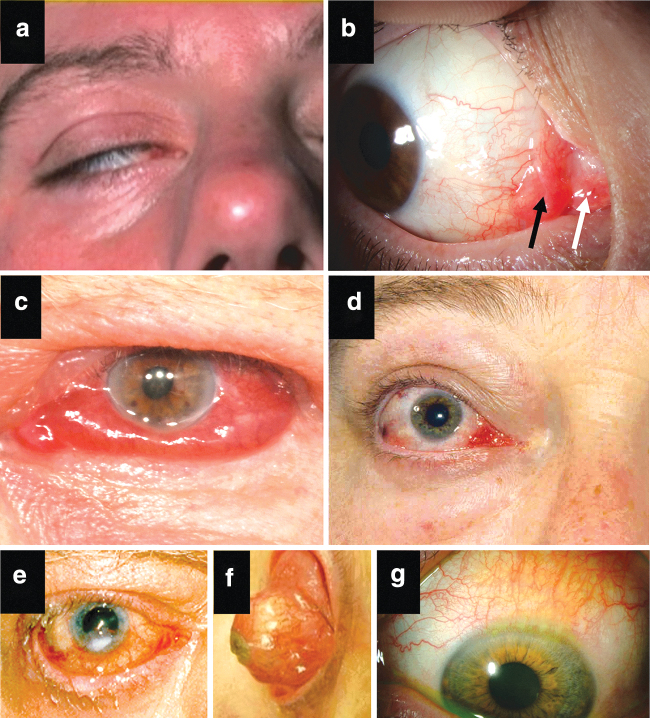
Composite of selected clinical features in patients with TED. Patient photographs provided with their consent demonstrate **(a)** lagophthalmos (inability to close eyelid completely); **(b)** edema and hyperemia of the caruncle (white arrow) and plica (black arrow) (courtesy of P. Perros); **(c)** chemosis (conjunctival edema) (courtesy of P. Perros); **(d)** lateral flare due to upper eyelid retraction (courtesy of P. Perros); **(e)** exposure keratopathy (courtesy of P. Perros); **(f)** globe subluxation. This is a rare complication in which the eye is displaced anterior to the retracted eyelids. Trapping of the globe may result in painful keratopathy or vision loss. This patient is seen at time of urgent surgery to decompress the orbits and narrow the lid aperture (courtesy of P. Dolman); **(g)** superior limbic keratoconjunctivitis in eye associated with marked upper lid retraction. This chronic recurring condition is often associated with thyroid disorders and is characterized by enlarged vessels and subepithelial edema involving the superior bulbar conjunctiva and corneal limbus (courtesy of P. Dolman).

A primary objective in the evaluation of TED is to assess factors that inform management and predict outcomes. There is an important distinction in TED between the two interdependent components of *inflammatory activity*, manifested by pain, redness, and edema, and disease *severity*, including proptosis, lid malposition, exposure keratopathy (Fig. 2e), impaired ocular motility, and optic neuropathy. The presence of multiple features of inflammation usually signifies active disease. A history of progressive TED further supports the presence of active disease. Definitions of activity and severity are given in Table 1.

When it is unclear whether the disease is active, repeating the assessments after an interval of 4–6 weeks will usually provide the answer, based on a measurable worsening in disease symptoms and signs. The small proportion of patients with TED who subsequently progress to sight-threatening disease can often be identified from the history and examination.^24,25^ These “high-risk” TED patients are characterized by the features given in Table 2. Such cases merit close follow-up.

**Table 2.** Characteristics of High-Risk Thyroid Eye Disease Patients

Background
Male sex
Age >50 years
Tobacco smoker
History
Unstable thyroid function
Diabetes mellitus
Radioiodine in the past 6 months
Progressive symptoms and/or signs of TED
Orbital aching
Diplopia
Examination
Marked soft tissue inflammatory features
Lagophthalmos (Fig. 2a)
Impaired ocular motility, particularly elevation

The features outlined are associated with an increased probability of developing sight-threatening TED.^24^

Endocrinologists should be familiar with basic elements of the eye examination for patients with TED as needed to grade severity and activity, according to the worst affected eye. Diagnostic criteria for TED as well as key elements of the eye examination for nonophthalmologists are reviewed in Supplementary Figure S3. A 5-minute patient assessment tool combining subjective and basic objective patient evaluation to diagnose TED and determine a need for ophthalmology referral was found to be efficacious in a pilot trial.^26^

The most widely used assessment of TED *activity* is the CAS, adopted by the EUGOGO^19^ and the ATA clinical practice guidelines on the management of hyperthyroidism.^18^ A 7-point CAS is currently favored for clinical evaluation that includes pain, erythema, and edema, whereas the 10-point version assesses change over time, using three additional points for worsening proptosis, motility, or visual acuity^27^ (Table 1). Advantages of CAS include its use of purely clinical parameters and moderate ability to predict response to immunomodulatory therapy.^27,28^

Examples of CAS elements with patient photographs are provided in open access at (https://onlinelibrary.wiley.com/doi/epdf/10.1046/j.1365-2265.2001.01349.x). Disadvantages include the binary (yes/no) classification in each category, assignment of equal weight to parameters with divergent clinical importance, and being prone to both false positive (congestive orbitopathy) and false negative predictions (aging and darker skin complexion) of response to treatment.^29,30^

Assessment of TED *severity* allows an appraisal of the patient's immediate or future threat to vision, a semiquantitative method for determining change over time, as well as for use in research to facilitate interstudy comparison and meta-analysis. Specific ophthalmic measures including visual acuity, ocular motility and alignment, proptosis, and lid retraction can be accurately documented along with their changes in the clinical assessment of TED severity. A widely used method for broadly categorizing TED severity recommended by EUGOGO^19^ classifies patients as having mild, moderate-to-severe, and sight-threatening disease (Table 1).

Certain clinical parameters indicate a higher risk for development of sight-threatening TED. Features suggesting a threat to vision include spontaneous orbital aching, diplopia, or restriction of eye movements and lagophthalmos (incomplete lid closure), evolving over a period of weeks or months (Fig. 2a).^24^ In addition, decreased visual acuity, color vision or visual field, a relative afferent pupillary defect (Marcus-Gunn pupil), and optic disk swelling or pallor are indicative of optic neuropathy. Along with the objective changes of the parameters that comprise severity of TED, its impact on daily living should be noted (see Section 4.2, on assessment of QOL).

A comprehensive assessment system for gauging both activity and severity is known as VISA (standing for vision, inflammation, strabismus, and appearance). The VISA Clinical Recording Form (https://thyroideyedisease.org/clinical-visa-recording-forms/) grades both disease severity and activity using subjective and objective inputs. It organizes the clinical measurements of TED into four severity parameters: V (vision, DON); I (inflammation, congestion); S (strabismus, motility restriction); and A (appearance, exposure).

A summary grade for each severity parameter is recorded at the end of the form so that directed therapy may be chosen based on the parameters involved.^30^ Activity is determined at the first visit by subjective progression in any VISA symptoms over the previous 2 months, or by documented worsening clinical measurements between visits.

**Key Point 4.1.1:** Endocrinologists should be familiar with basic elements of a TED examination enabling assessment of both activity and severity.

**Key Point 4.1.2:** Assessment of patients with TED should include activity, severity (with particular attention to impaired ocular motility and visual loss), trend across time, and impact on daily living.

### 4.2. Assessment of QOL

TED has major negative effects on QOL.^31^ Impairment in function may negatively impact daily activities (reading, driving, computer work, and watching television), as well as result in dry eye, photophobia, and retro-orbital pain.^31^ Changes in appearance may lead to psychosocial disability.^32–34^ In general, the negative effects on QOL correlate with activity and severity and may persist for years.^35^ The impact of TED on QOL also depends on the specific cultural and psychosocial circumstances of each individual patient and is an important parameter that influences decisions about treatment. Furthermore, the risk-to-benefit ratio of the proposed therapeutic choices should fully encompass the disease impact on the patient's QOL. A widely used and validated QOL instrument is the GO-QOL.^31^

**Key Point 4.2.1**: The physical and psychosocial impact of TED should be assessed for each patient, as it informs treatment decisions. When formal quantification of QOL is deemed appropriate, GO-QOL is the preferred instrument.

### 4.3. Formal ophthalmology evaluation

Ophthalmologists with expertise in TED can confirm the diagnosis and assess severity, activity, and disease trajectory to help plan management. Historical features portending a more severe TED course with diplopia or DON are listed in Table 2.^36^ A recent onset with rapidly worsening symptoms predicts aggressive disease, requiring expert evaluation, close follow-up, and prompt intervention.^37^

The directed ophthalmic examination uses standardized techniques to document how the orbit, eye, and eyelids are affected by TED.^38^ General ophthalmologists can assess vision, ocular motility, and the structures of the eye, and distinguish vision loss from various possible sources, including DON, corneal exposure, astigmatism, or choroidal folds. A subspecialist in oculoplastic and orbital disease will be able to differentiate TED from other orbital conditions, assess imaging, participate in medical management, and perform surgical interventions.

Table 3 organizes the functional and anatomic changes into four clinical categories (vision, soft tissue changes, impairment of ocular motility, and structural changes [proptosis and eyelid malposition]), and lists available ophthalmic techniques and ancillary tests used to assess them.^39^ For each finding the clinician must consider TED-related causes, non-TED-related causes, or both.

**Table 3.** Formal Ophthalmic Examination for Thyroid Eye Disease Based on Vision, Inflammation, Strabismus, Appearance

	Clinical ophthalmic examination	Ancillary eye tests	TED-associated mechanisms	Non-TED-associated causes
Vision Central vision Color vision Peripheral vision	Snellen chart Color plates Pupil testing Fundus examination	Pattern visual evoked response Optical coherence tomography (analyzes optic nerve for nerve fiber loss) Visual field Corneal topography	DON Corneal exposure Dry eye Choroidal folds	Cataract Macular disease Glaucoma Diabetic retinopathy
Inflammation (soft tissue changes) Redness and swelling of eyelids and conjunctiva	Slit-lamp biomicroscope	Clinical photographs EUGOGO	Inflammation Venous congestion Superior limbic keratoconjunctivitis (Fig. 2g)	Allergic infective conjunctivitis Iritis or scleritis Dural cavernous fistula Eyelid margin disease Eyelid infection or neoplasia Orbit neoplasia Orbit inflammation
Strabismus (ocular motility changes) Diplopia Ductions Strabismus	Corneal light reflex test (Supplementary Fig. S1a, b) Cover testing	Orthoptics examination: Perimetric ductions Field of binocular single vision (area of binocular gaze with single image) Fresnel prism Prism measurements	Extraocular muscle restriction	Myasthenia gravis Dural cavernous fistula Orbital myositis Orbital lymphoma Orbital metastasis IgG4 disease Cranial nerve III, IV, VI palsy
Appearance (structural changes) Lid retraction	Ruler measure Marginal reflex distance (the distance between the upper lid margin and the corneal reflex when the eye is in the primary position)	Clinical photographs	Upper lid retraction Levator scarring Compensatory levator Retraction from restricted IR muscle Lower lid retraction From proptosis From IR recession surgery	Lid retraction from Orbital fracture Maxillary sinus atelectasis
Proptosis	Exophthalmometry		Fat expansion Muscle enlargement GC-induced lipogenesis	Orbital neoplasia Inflammation Hemorrhage/trauma GC-induced proptosis
Corneal exposure	Slit-lamp biomicroscope Fluorescein stain		Lid retraction Lacrimal gland inflammation	Dry eyes Corneal infection Eyelid margin disease

EUGOGO, European group on Graves' orbitopathy; GC, glucocorticoid; IR, inferior rectus.

Visual impairment may be documented by measuring central visual acuity, color perception, and peripheral vision. Dry eyes and corneal exposure impairing vision are identified with the slit-lamp biomicroscope. Features of DON and their prevalence at presentation include color desaturation (98% of DON patients miss two or more plates), central vision loss (90% record 20/40 or less), and relative afferent pupillary defect (Supplementary Fig. S1d) (50%).^24^ Optic disk edema, hyperemia, or atrophy is rare in DON and their absence does not reduce suspicion or eliminate a diagnosis of DON.^24^ Perimetry may show visual field defects consistent with optic nerve compression, which might be missed on fundoscopy alone.^40^

Eyelid and conjunctival edema and redness result from inflammation, corneal exposure, or congestion, and are best assessed with the slit-lamp.^41^ Rarely, in severe cases, globe subluxation develops, presenting as the equator of the globe protruding beyond the retracted lids (Fig. 2f). Chronic orbital congestion, resulting from impaired venous drainage, may occur independent of active inflammatory changes. Grading is more reliable with clinical photographs or the EUGOGO atlas.^42^

Restriction of eye movements (ductions) from fibrotic or “tight” EOMs leads to diplopia, typically in upward and lateral gaze. Diplopia is graded from 0 to 3 using the Gorman score (absent, intermittent, inconstant, or constant). Ductions are measured with the light-reflex method (reliable to within 12 prism diopters) (Supplementary Fig. S1a, b).^43^ Strabismus (ocular deviation) is measured with prisms. An orthoptic evaluation aids in prism fitting and surgical planning.^39^

Over 90% of TED patients develop upper eyelid retraction. Proptosis is the second most common finding and is measured with the exophthalmometer (Supplementary Fig. S1c); intraobserver reliability with this device is usually within ±1 mm.^44^ The combination of eyelid retraction and proptosis may lead to corneal exposure, best assessed with the slit-lamp. Upper eyelid retraction is also a feature of thyrotoxicosis of any cause and thyroid status needs to be considered when assessing the position of the upper lids.

Ophthalmological measurements are necessary to fully assess severity and activity of TED. On each follow-up visit, repeat evaluations allow assessment of the disease course (worse, stable, or improving) and response to therapy. This may be facilitated by using a standardized clinical recording form (such as the VISA or EUGOGO forms, downloadable at *thyroideyedisease.org* or *eugogo.eu*), which organize the clinical data to permit easy review and comparison between visits.

### 4.4. Imaging

**FIG. 3.**
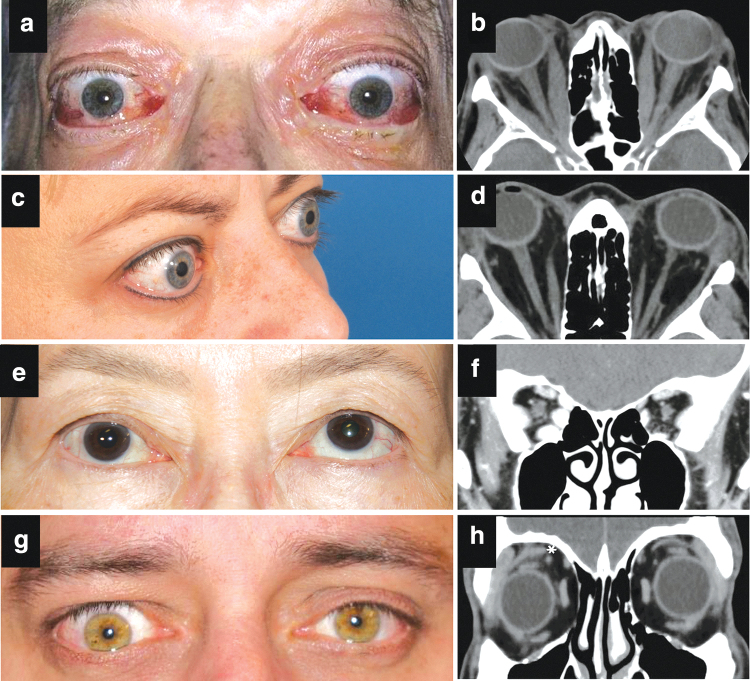
Composite clinical–radiographic correlation in patients with TED. Clinical and radiographic image correlations provided with patient consent (courtesy of P. Dolman): **(a, b)** extraocular muscle enlargement causing periorbital soft tissue congestion, ocular motility restriction, and optic nerve compression with dysthyroid optic neuropathy; **(c, d)** proptosis in a patient with TED and predominant retroocular fat compartment expansion; **(e, f)** restricted upward gaze on the right due to right inferior rectus muscle enlargement and fibrosis; **(g, h)** right upper eyelid retraction and lateral flare due to enlargement and fibrosis of the right levator palpebrae superioris muscle (asterisk).

Orbital imaging is not mandatory for patients with bilateral TED but should be considered in the following situations: (1) to exclude other diagnoses in atypical cases, such as unilateral or euthyroid disease; (2) to assist with assessment in severe cases, in identifying apical crowding, a risk for DON (Fig. 3a, b); (3) to prepare for orbital surgery and in some cases for strabismus surgery (Table 4). Both CT and MRI identify orbital tissue enlargement, including EOMs, orbital fat, and lacrimal glands.^45,46^

**Table 4.** Primary Indications for Imaging in Suspected or Confirmed Thyroid Eye Disease

Exclusion of other diseases in atypical TED
Euthyroid, without history of thyroid dysfunction
Clinically unilateral or markedly asymmetric
Absent upper lid retraction
Upper lid ptosis
Atypical strabismus
Severe orbital pain
Assessment in confirmed TED
Sight-threatening TED
Planning of orbital and in some cases strabismus surgery

Proptosis related to fat compartment expansion alone, without EOM enlargement, can be demonstrated with imaging (Fig. 3c, d). EOM enlargement is typically fusiform with sparing of the tendons and involves, with decreasing frequency, the inferior and medial recti (Fig. 3e, f), superior rectus, or, rarely, all recti and oblique muscles. Levator enlargement as a source of eyelid retraction (Fig. 3g, h) is visible on orbital CT.^47^

The standard imaging modality is noncontrast CT scan, which is inexpensive, readily available, and allows assessment for decompression surgery. Occasionally, contrast CT is preferred as it shows enhancement of the involved EOM and surrounding fat as an indicator of acute inflammation and may be valuable when a diagnosis other than TED is suspected. MRI provides excellent soft tissue resolution and identifies edema within the muscle on T2 or Short-Tau Inversion Recovery sequence suggesting active disease, but at greater expense, longer imaging duration, and poor definition of the bony walls.^45^

Other imaging modalities are mainly used in research. When clinical and radiological findings are inconsistent with TED, tissue biopsy of an involved muscle must be considered for exclusion of other pathologies.^48^ Repeat imaging in patients with TED is generally not required except for the development of new signs or postoperative complications.

**Key Point 4.4.1:** Orbital imaging using contrast-enhanced CT or MRI is preferred for atypical or severe cases of TED to help determine activity and to exclude other etiologies that could be confused with TED.

**Key Point 4.4.2:** Noncontrast CT is the preferred modality in patients with TED who are being considered for surgery.

## 5. OVERALL APPROACH TO THERAPY

### 5.1. Local and lifestyle measures

In addition to optimally controlling hyperthyroidism as described in clinical practice guidelines,^4,18^ some nonsystemic treatments and lifestyle measures can be beneficial in TED. Dry eye is common and is caused by corneal exposure and lacrimal gland dysfunction. Corneal exposure occurs due to lid retraction and lagophthalmos (Fig. 2a). Dry eye syndrome (DES) can be treated with artificial tears containing either sodium hyaluronate or carboxymethylcellulose.^49^ Bland nonmedicated lubricating eye drops, gels, or ointment can be used at night, along with taping of the lids in patients with lagophthalmos, or wearing a headband tightened over a vaseline-moistured eye pad.

Head of the bed elevation, such as sleeping with additional pillows, is sometimes used to relieve edema. Photophobia can be a consequence of DES and is frequently managed with dark glasses and lubricants. Diplopia can be improved with selective ocular occlusion or with Fresnel press-on prisms. Patients should abstain from smoking and avoid second-hand smoke exposure.^50^

Local and lifestyle measures and watchful monitoring will be sufficient in the majority of patients with mild disease, which in due course will remit completely or partially.^51^ In selected patients with moderate-to-severe TED, a “watchful monitoring” strategy may also be acceptable. Placebo-controlled studies have shown a 10–59% chance of spontaneous disease inactivation and improvement in proptosis and diplopia in patients who satisfied study criteria for treatment (Table 5).

**Table 5.** Efficacy of Pharmacological Therapy for Active Moderate-to-Severe Thyroid Eye Disease

A. Comparisons of outcomes from baseline to after treatment^a,b^					
Drug (ref)	Composite outcome (%)	Clinical activity score (%)	Proptosis (%)	Diplopia (%)	Disease relapse (weeks)
IVGC^67,68,71,72^	23–53	45–83	0–46	0–19	21–40% (week 12)
MMF+IVGC^68^	63	80	No change	No change	8% (week 12)–11% (week 24)
RTX^100^	8	31	No change	No change	15% (week 40)
RTX^67^	60	100	No change	No change	0% (week 40)
TEP^91^	74	62	77	70	29% (week 51)–37% (week 27- see text)
TCZ^112^	73	93	27	7	No data
Placebo^91,100,112^	10–22	22–59	No change	No change	0 (week 12)–8% (week 51)

B. Comparisons of treatment outcomes between groups					
Drug (ref),* n* = no. of randomized	Composite outcome	Clinical activity score	Proptosis	Diplopia	Disease relapse (weeks)
MMF vs. GC^106^ IVGC *n* = 78, MMF *n* = 80	Favored MMF 79% vs. GC 51%	Favored MMF 94% vs. 69%	Favored MMF 69% vs. GC 40%	Favored MMF 90% vs. GC 64%	Favored MMF 0% vs. IVGC 6%
MMF+IVGC vs. IVGC^68^ MMF+IVGC *n* = 76, IVGC *n* = 76	No difference between groups	No difference between groups	No difference between groups	No difference between groups	No difference between groups
	Post hoc MMF+IVGC 67% vs. IVGC 46%				
OGC vs. IVGC^74^ IVGC *n* = 35, OGC *n* = 35	Favored IVGC 77% vs. OGC 51%	Favored IVGC 77% vs. OGC 51%	Favored IVGC 60% vs. OGC 40%	No difference between groups	Favored IVGC 0% vs. OGC 11% (week 24)
RTX vs. IVGC^67^ RTX *n* = 15, IVGC *n* = 16	Favored RTX 60% vs. IVGC 38%	Favored RTX 100% vs. IVGC 69%	No difference between groups	No difference between groups	Favored RTX 0% vs. IVGC 31% (week 76)
RTX vs. placebo^100^ RTX *n* = 13, placebo *n* = 12	No difference between groups	No difference between groups	No difference between groups	No difference between groups	No differences between groups (week 50)
Statin+IVGC vs. IVGC^71^ IVGC, *n* = 39, IVGC+statin *n* = 41	Favored atorvastatin+IVGC 51% vs. IVGC 28%	No difference between groups	No difference between groups	No difference between groups	Favored atorvastatin+IVGC 0% vs. IVGC 15%, *p* = 0.011) (week 24)
TEP vs. placebo^91^ TEP *n* = 84 placebo *n* = 87^c^	Favored TEP 74% vs. placebo 14%	Favored TEP 62% vs. placebo 22%	Favored TEP 77% vs. placebo 15%	Favored TEP 70% vs. placebo 31%	Data only for TEP 29.4–37% (weeks 27–51)^d^
TCZ vs. placebo^112^ TCZ *n* = 15, placebo *n* = 17	Favored TCZ 93% vs. placebo 59%	Favored TCZ 73% vs. placebo 29%	Favored TCZ^e^	No difference between groups	No data provided

^a^ Comparisons of efficacy between treatments are subject to bias due to heterogeneity of patient populations, assessment methodology, end points, definitions of response and relapse, and duration of follow-up. The composite outcome is a combination of activity and severity measures and is variably defined. Proptosis improvement was defined as a reduction ≥2 mm in most studies. Diplopia was assessed using the Gorman scoring system.

^b^ The figures in A represent statistically significant changes compared with baseline, unless marked “no change.”

^c^ Pooled data from two randomized controlled trials.^89,90^

^d^ Data for “flares”/relapses available for TEP group only (not placebo group).

^e^ Proptosis change from baseline TCZ −1.5 mm versus placebo 0.0 mm.

IVGC, intravenous glucocorticoids; MMF, mycophenolate mofetil; OGC, oral glucocorticoids; RTX, rituximab; TCZ, tocilizumab; TEP, teprotumumab.

**Key Point 5.1.1:** Local ocular measures and lifestyle intervention should be offered to all patients with TED. Lubricants and nocturnal eye masks may be used to prevent or treat corneal exposure. Ocular occlusion and prisms may be offered to relieve diplopia. The importance of smoking reduction or cessation should be explained, and smokers offered support for this goal.

### 5.2. Overview of systemic medical and surgical treatments for TED

Decisions concerning treatment beyond local measures are guided by a number of factors including patient symptoms, QOL, disease activity and severity, risk of deterioration, duration of TED, patient age and comorbidity, and patient preference.^52^ Sight-threatening TED requires urgent treatment, close monitoring of response, and often multimodal treatments.^24^ In general, treatments during the active phase of TED are aimed at suppressing inflammation and preventing complications and are largely medical. Immunomodulatory treatments are most effective in patients with short duration of TED, the optimal being <6–9 months.^53,54^

Surgical rehabilitation for proptosis (Supplementary Fig. S2c, d), chronic congestion (Supplementary Fig. S2a, b), strabismus, or lid malposition (Supplementary Fig. S2g, h and i, j) is typically delayed until the quiescent phase,^55–57^ although urgent surgery may be necessary during the active/progressive phase for DON, severe corneal exposure, or globe subluxation. Systemic medical and surgical treatment for TED are discussed in Sections 6–8.

### 5.3. Setting for TED care

Optimal management of moderate-to-severe and sight-threatening TED requires a collaborative approach from endocrinologists and ophthalmologists (Section 3.5). Infusion centers, where immunomodulatory therapy may be safely delivered in a controlled setting, vary widely from one institution to the next, but share the common elements of an ability to monitor for and respond rapidly to infusion-related AEs.

**Key Point 5.3.1:** Input from both endocrinologists and ophthalmologists with TED expertise is recommended for optimal management in patients with moderate-to-severe and sight-threatening TED.

### 5.4. Referral to ophthalmology

**FIG. 4.**
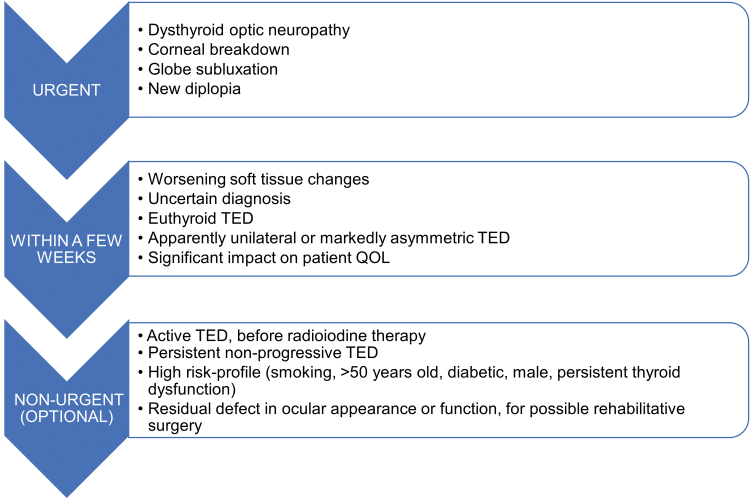
Referral guidance for patients with TED. Suggested criteria and timing for ophthalmological examination vary according to the clinical presentation of the eye disease (see Section 5.4).

Endocrinologists managing patients with TED should consider referring them for TED specialty care (as defined in Section 2.1 and Section 3.5). Suggested criteria and timing for ophthalmological referral vary according to the clinical presentation of the eye disease, as summarized in Figure 4. The referring endocrinologist will help the ophthalmologist by direct communication, explaining the pertinent clinical features, thyroid status, and risk factors as well as the urgency of referral.

**Key Point 5.4.1:** An ophthalmologist should be consulted when the diagnosis of TED is uncertain, in cases of moderate-to-severe TED, and when surgical intervention needs to be considered. Urgent referral is required when sight-threatening TED is suspected or confirmed.

## 6. THERAPY FOR MILD TED

### 6.1. Medical therapy for mild TED

Selenium has been recommended for patients with mild TED.^19^ The rationale for the use of selenium centers around its incorporation into selenocysteine-containing proteins, which may have antioxidant and immunomodulatory effects on orbital inflammation.^58^ In a blinded placebo-controlled multicenter trial conducted in Europe, including geographic areas of marginal dietary selenium intake, patients were randomized to receive 100 μg of selenium selenite twice daily, or placebo for 6 months.^59^ After 6 months of therapy, improvements in CAS as well as in GO-QOL scores were noted with selenium therapy, but not with placebo, and persisted for an additional six months after therapy was stopped.

Overall, patients treated with selenium were more likely to have improvements in their TED, and less likely to have disease progression.^59^ Based on the results of this trial, a 6-month course of selenium therapy is recommended for treatment of mild GO of relatively short duration by the EUGOGO,^19^ and the ETA.^4^ There is no evidence that selenium provides benefit in patients with moderate-to-severe TED. Selenium selenite contains ∼45% elemental selenium by weight.

Whether selenium therapy is efficacious in selenium sufficient parts of the world remains an important open question. The U.S. recommended daily allowance for selenium is 55 μg daily,^60^ which is far less than the dose used in mild TED. The potential benefits of selenium supplementation should be balanced against the possible risks of AEs (e.g., possible increased risk of prostate cancer and squamous cell cancers, and type 2 diabetes, though controversial),^61^ and current evidence does not support extending the duration >6 months.

**Key Point 6.1.1:** A single course of selenium selenite 100 μg twice daily for 6 months may be considered for patients with mild active TED, particularly in regions of selenium insufficiency.

### 6.2. Surgery for minimal changes in proptosis and lid retraction

Although mild TED is traditionally defined as having insufficient impact on daily life to warrant immunomodulatory or surgical intervention, even minimal proptosis or lid retraction may project an angry or anxious look, and eyelid fat bulges may give the appearance of premature aging to the face. For some individuals these changes negatively impact their self-confidence and social functioning. Individualized corrective procedures include eyelid narrowing to correct retraction, and blepharoplasties to tighten loose skin and remove fat bulges. The sequence and type of surgery are chosen based on the severity of the changes, the goals of the patient, and the known procedural risks. The indications, timing, and complications of surgery for TED are discussed in Sections 7–8.

**Key Point 6.2.1:** The clinician should regularly assess the psychosocial impact of concerns about appearance.

## 7. MANAGEMENT OF MODERATE-TO-SEVERE TED

### 7.1. Medical therapies

A range of therapies are available for treatment of moderate-to-severe active TED, as supported by evidence from RCTs. Efficacy and safety are key elements in deciding among available therapies. Several therapies require parenteral infusion and premedication to avoid common AEs. Serious AEs can rarely occur during infusion and beyond, making it imperative that these therapies are administered in a safe environment. Individual patient features are important as some treatments are more effective for specific components of TED than others (Table 5).

**FIG. 5.**
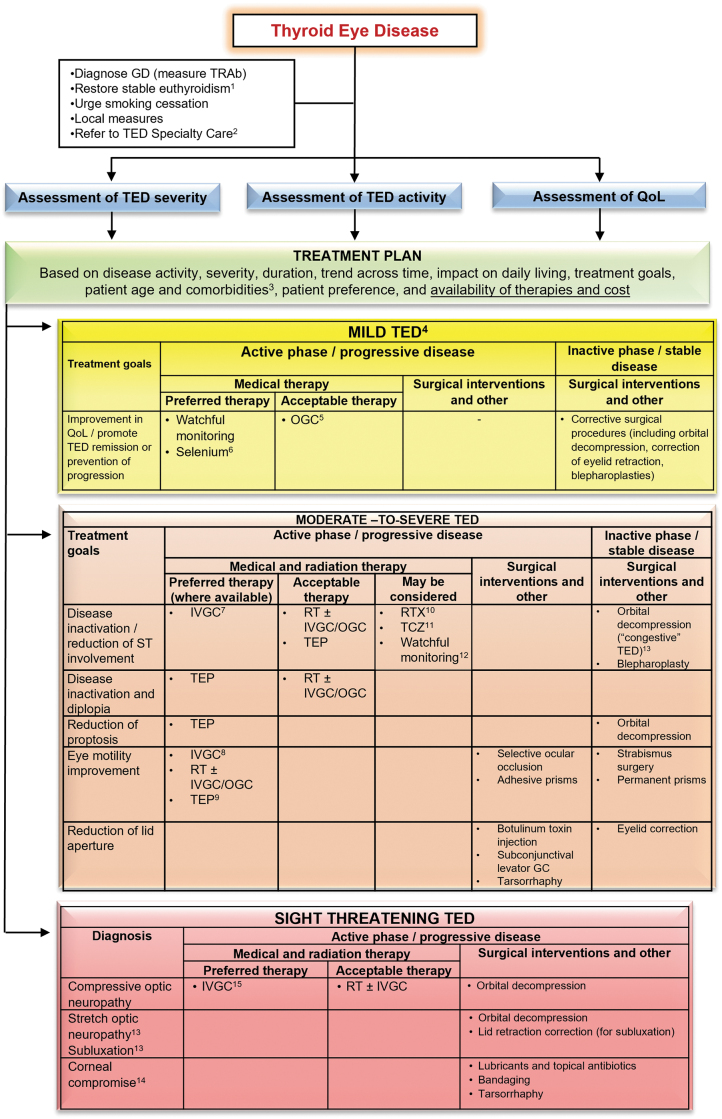
Overview of the management of TED. An individualized approach to the management of TED, based on disease activity, severity, duration, trend across time, impact of the disease on daily living, treatment goals, patient age, and comorbidities, as well as the availability and relative costs of therapies, must be advised. Wherever possible, the task force members ranked therapeutic approaches as either “preferred,” “acceptable,” or “may be considered” (see Section 2.1. for definitions). ^1^See Figure 1. ^2^Except for the mildest cases improving with local measures. ^3^See Table 8. ^4^In most patients with mild TED, a “watchful monitoring” strategy is sufficient (it includes simple measures, see Section 5.1 and Fig. 1). Selected cases (with a significant decrease in QOL) may be treated as moderate-to-severe TED. ^5^In patients with symptomatic inflammatory soft tissue involvement or if radioactive iodine is used (oral glucocorticoids prophylaxis). ^6^Particularly in countries that are selenium insufficient. ^7^Standard treatment—IVGC (cumulative dose 4.5 g). ^8^In selected patients, a higher cumulative dose of methylprednisolone (7.5 g) may be considered. ^9^In patients with prominent soft tissue involvement and diplopia. ^10^In patients with a short duration of TED (< 9 months). ^11^In patients who are intolerant or resistant to IVGC. ^12^In selected patients with moderate-to-severe TED, a “watchful monitoring” strategy may be acceptable. ^13^See Section 7.3.2, and Supplementary Figure S2a, b. ^14^If there is coexistent active disease, then medical treatment as for moderate-to-severe disease is indicated in parallel with surgical treatment. ^15^High doses of IVGC (500–1000 mg of methylprednisolone) for 3 consecutive days or on alternate days during the first week. IVGC, intravenous glucocorticoid.

Appraising the role of different medical therapies is limited by heterogeneity in inclusion criteria (particularly disease activity and duration of TED), and in methods for assessing response to treatment as well as documenting and classifying AEs. The introduction of biologics has raised the cost of treatment many fold over conventional agents. No cost-effectiveness appraisals are available, nor comparative effectiveness trials for any currently available medical therapy for TED. The use of standardized treatment outcomes in clinical trials involving patients with TED has been recently proposed.^62^ Making decisions about treatment of moderate-to-severe TED lends itself particularly well to the principles of shared decision making. Tables 5–8 and Figure 5 are intended to aid this process.

**Key Point 7.1.1:** Infusion therapies for TED should be administered in a facility with appropriate monitoring under the supervision of experienced staff. Awareness and surveillance for adverse side effects are recommended throughout the treatment period.

**Key Point 7.1.2:** Clinicians should balance the demonstrated efficacy of recently introduced therapies against the absence of experience on sustained long-term efficacy, safety, and cost-effectiveness.

#### 7.1.1. Glucocorticoids

##### Mode of action

GCs alter the distribution, survival, and trafficking of leukocytes, interfere with the function of B and T cells, and reduce recruitment of monocytes and macrophages.^63^

##### Clinical experience

GCs have been used for >60 years for TED and studied extensively. RCT data have been published on oral glucocorticoid (OGC)^64–66^ and intravenous GC (IVGC)^65–68^ from >300 to 500 patients, respectively. Data on IVGC AE in TED are documented for >1200 patients treated.^65^

##### Efficacy

There is only one small RCT comparing IVMP with placebo in 16 patients with TED,^69^ which showed beneficial effects. Data pertaining to the efficacy of IVGC is largely derived from RCTs in which IVGC is compared with other therapies such as OGC,^64,70^ RTX,^67^ or to combination therapy including mycophenolate mofetil plus IVGC^68^ or IVGC plus atorvastatin.^71^ In addition, a large RCT comparing three different cumulative doses of IVGC provides data on the efficacy of this modality, discussed hereunder.^72^ Although several nonrandomized studies on GC have been performed,^73^ this section emphasizes data from relevant RCTs.

###### Activity

Improvement in disease activity, defined variably, occurs in 58–83% of IVGC-treated patients,^67,72,74^ compared with 51% of those treated with OGC.^74^ An RCT including 70 patients with active moderate-to-severe TED showed improvement in median CAS values from 5 to 2 with IVGC, versus improvement in CAS values from 5 to 3 in OGC-treated patients.^74^ Overall, 77% (27/35) of patients treated with IVGC and 51% (18/35) of those treated with OGC experienced improvement in CAS by 3 points. The IVGC arm of another RCT included 81 patients with active moderate-to-severe TED in whom CAS fell from a baseline mean of 3.66 and 3.66 (right and left eyes) to 1.65 and 1.68, respectively, at 36 weeks.^68^

Another RCT that included an IVGC treatment arm in 16 patients with TED found that 75% had CAS improvement by ≥2 at 24 weeks, and 69% had CAS inactivation to values <3.^67^ A recent RCT comparing IVGC plus atorvastatin with IVGC alone found that 28% of 39 patients treated with IVGC alone had improvement in a composite outcome.^71^ Finally, in an RCT involving 159 patients with active moderate-to-severe TED, comparing three IVGC cumulative doses of 2.25, 4.98, and 7.47 g, improvement in CAS >2 points was found at 12 weeks in 81–83% using the two higher dose regimens and 58% of the low-dose treated patients.^72^ Disease inactivation (defined in this study as CAS ≤2) occurred in 45–65% of patients.

###### Severity

Proptosis is reported to improve by *>*2 mm at 12 weeks in 20–60% of patients,^72,74^ but studies with longer term follow-up show no proptosis response.^67,68^ With regard to diplopia, a range of responses to IVGC have been reported from little overall improvement^68^ to 57% reduction in constant diplopia at 12 weeks.^74^ A comparison of IVMP doses showed that a high cumulative dose (7.5 g) was associated with modest improvement in ocular motility (elevation and abduction) in 33% of patients receiving this dose, with no difference in subjective diplopia compared with lower cumulative doses.^72^ A recent meta-analysis found only small improvements in proptosis and diplopia compared with baseline.^75^

###### Quality of life

QOL assessments have shown variable improvement from baseline for IVGC.^68,72,74^ A 2005 study utilizing the SF-36 to assess physical and psychological components of QOL found that an overall rating of good or excellent occurred in 9% of patients at baseline but improved significantly to 80% after therapy.^74^ A study utilizing the GO-QOL tool has shown improvement of at least 6 points on a 100-point scale in 48–67% of patients after three different cumulative doses of IVGC.^72^ Results from the IVGC arm of another trial showed improvement of 5–10 points at 24 and 36 weeks compared with baseline.^68^

##### Dosing and route of administration

Dosing of IVMP was tested in a large RCT (*n* = 159) comparing three doses with a finding that a cumulative dose of 4.5 g (administered as 0.5 g weekly × 6 weeks followed by 0.25 g weekly for an additional 6 weeks) was judged to be suitable for most patients with moderate-to-severe TED for disease inactivation.^72^ Topical GC drops are rarely helpful in TED, and retrobulbar GC injections pose risks of injury to the globe and are less effective than systemic GC.^76^

##### Nonresponse and relapses after completion of treatment

Failure to inactivate TED is observed in 20–40% and 40–60% of patients treated with IVGC or OGC, respectively.^72,74^ Relapse after treatment with different doses of IVMP was studied in a large multicenter study but limited to 12 weeks of follow-up after completion of treatment.^72^ In this study, relapse, or deterioration, as defined by either the development of DON or at least two additional items among the following: widening palpebral fissure, an increase in soft tissue inflammatory changes by two grades on the NOSPECS system,^19^ worsening proptosis by ≥2 mm, or increasing restriction in eye movement and/or worsening diplopia, occurred in 31% of patients.

New DON and worsening diplopia can occur despite improvement in inflammation with GC therapy, with DON occurring in 25 of 144 (17%) patients a mean of 5.5 months after starting GC in one retrospective analysis.^77^ Early TED deterioration^78^ or unresponsiveness^79^ after 6–8 weeks of IVGC may predict treatment failure and alternative therapies should be considered.

###### Safety

AEs in relation to IVGC have been reviewed from the published literature relating to a total of 1220 patients.^65^ A systematic review found that 43 of 101 (42.6%) patients treated with IVGC for TED developed a total of 119 AEs, including 2 events (1.7%) considered major (hepatitis and depression), 49 (41%) moderate, and 68 (57%) classified as minor.^73^ The risk of death in this study was 0.6%, resulting from cardiovascular and hepatic causes. Common AEs include new or worsened hyperglycemia, worsening hypertension, weight gain, Cushingoid appearance, increased intraocular pressure, insomnia, depression, and psychosis.^68,72,74^

Major AEs were noted in 6.5% of patients in another large study and are more frequent with higher cumulative doses.^72^ A cumulative dose of >8.0 g IVMP is associated with a risk of severe hepatotoxicity,^73,80,81^ and should be avoided. Whether this risk dissipates after a time interval and whether OGCs add to the risk are unknown. The decision to exceed this limit, as in cases of new onset DON, should take careful account of expected benefits balanced against the risks for the individual patient as well as consideration of alternative treatment modalities.

Exclusion of viral hepatitis (by testing for viral DNA) and occult infection, such as tuberculosis, is needed before treatment, particularly for patients with a high risk of such infections. Monitoring for side effects during therapy (Table 6) is required. Contraindications to therapy include active viral hepatitis and hepatic dysfunction, severe cardiovascular disease, uncontrolled hypertension or diabetes, and untreated psychiatric disorders.^19^

**Table 6.** Adverse Effects of Medical Therapy for Thyroid Eye Disease

Drug (ref)	Frequency (%)	Severity^a^			
		Minor (Grade 1)	Moderate (Grade 2)	Severe (Grade 3)	Life threatening (Grades 4–5)
IVGC^68,72,74^	≥10		Hyperglycemia		
	5–9.9	GI symptoms		Infection	
	1–4.9	Flushing	Hypertension depression Weight gain	Psychosis	
	<1				Death, hepatic necrosis, myocardial infarction, stroke
OGC^66,74^	≥10	GI symptoms	Hyperglycemia, weight gain, Cushingoid facies		Not reported
	5–9.9		Hypertension	Infection	
	1–4.9		Depression		
MMF^106^	1–4.9			Infection, hepatitis	
MMF+GC^68^	≥10	GI symptoms		Infection	
	5–9.9				
	1–4.9		Sleep disorder		
RTX^53,67,100^	≥10		Infusion reaction (nonsevere)		
	5–9.9	GI symptoms		Transient visual loss^b^	
	1–4.9			Vasculitis	
	<1				Infusion reaction (severe)
TEP^89–91^	≥10	GI symptoms, myalgias, alopecia, fatigue	Hyperglycemia	Hearing loss, inflammatory bowel disease aggravation	
	5–9.9	Dry skin	Taste disturbance		
	1–4.9				Cerebral hemorrhage
TCZ^84,111,112^	≥10	Fatigue	Hyperlipidemia, neutropenia	Infection	
	5–9.9	Pruritus		Hepatitis	
	1–4.9		Thrombocytopenia Transaminase elevation		
	<1				Anaphylaxis Bowel perforation^c^

^a^ National Cancer Institute Common Terminology Criteria for Adverse Events (ctep.cancer.gov/protocoldevelopment/electronic_applications/docs/CTCAE_v5_Quick_Reference_8.5x11.pdf). Grade 1: Mild, asymptomatic or mild symptoms, clinical or diagnostic observations only, intervention not indicated. Grade 2: Moderate; minimal, local, or noninvasive intervention indicated; limiting age-appropriate instrumental ADL. Grade 3: Severe or medically significant but not immediately life threatening; hospitalization or prolongation of hospitalization indicated; disabling; limiting self-care ADL. Grade 4: Life-threatening consequences, urgent intervention indicated. Grade 5: Death related to adverse event.

^b^ Believed related to cytokine release syndrome.

^c^ Observed in other studies (not described in TED studies).

ADL, activities of daily living; GI, gastrointestinal; PML, progressive multifocal leukoencephalopathy.

###### Cost

OGC and IVGC are the least costly systemic treatments for TED (Table 7).

**Table 7.** Logistics of Medical Therapy for Thyroid Eye Disease

Drug	Route	Frequency and duration	Total drug cost/full treatment (Euros and U.S. dollars)		Ratio of cost of full treatment with drug over cost of full treatment with IVGC^a^		Impact of drug on vaccinations^b^
			€	$	€	$	
IVGC	IV	0.5 g weekly for 6 weeks, followed by 0.25 g weekly for 6 weeks	€70.0	$172	1	1	Decreased efficacy of vaccine; live vaccines deferred for 1 month after drug discontinuation
OGC	PO	Daily for 3 months (starting with 100 mg prednisolone daily, then tapering dose, cumulative dose 4 g)	€73.6	$440	1	3	Decreased efficacy of vaccine; live vaccines deferred for 1 month after drug discontinuation
MMF	PO	0.72 g daily for 24 weeks	€411	$1,191	6	7	Possible decreased efficacy of vaccine but data are controversial
RTX	IV	1 g two doses 1 weekly for 2 weeks	€4,308	$19,636	62	114	Decreased efficacy of vaccine; defer vaccination post-therapy until after B cells recovery
		0.5 g single dose	€1,698	$4,914	24	29	
		0.1 g single dose	€338	$990	5	6	
TEP	IV	Every 3 weeks for 6 months (first dose 10 mg/kg, subsequent doses 20 mg/kg, total number of infusions eight)	Not licensed in Europe	$357,997 for a 75 kg patient	5110	2080	Unknown
TCZ	IV	8 mg/kg every 4 weeks for 12 weeks (four doses)	€4,266	$14,519	61	84	Decreased efficacy of vaccine

^a^ Note on relative pricing—A course of IVGC costs €70.0 in Europe and $172 in the United States, derived from (www.pharmacychecker.com). These costs reference the price of medication alone, excluding administration costs. Figures are EU average costs supplied by manufacturers (Roche global) and approved by EMA (personal communication with one of coauthors).

^b^ Best to complete vaccination series at least 1 month before initiation of all these agents. Data about the impact of various drugs on vaccines are mainly derived from the literature on their use in rheumatological disorders.

IV, intravenous; PO, oral dosing.

**Table 8.** Clinical Situations That Favor a Particular Modality as Treatment for Active Moderate-to-Severe Thyroid Eye Disease

Clinical situation	IVGC/OGC	MMF^a^	RT	RTX	TEP	TCZ
Patients unresponsive or intolerant to GC	** =** ^b^	**?**	**√**	**√** /**√√**^c^	**?**	**√**
Adult patients <35 years of age	**√**	**√** ^d^	**X**	**√** ^d^	**√** ^d^	**√** ^d^
Chronic infection^e^	**X**	**X**	**√**	**X**	**√** /**√√**	**X**
Liver disease	**!/X**	**!**	**√**	**√**	**√** /**√√**	**!/X**
Active gastrointestinal disease	**!**	**!**	**√**	**!**	**!/X**	**!/X**
Cardiovascular disease	**!/X**	**√**	**!/X** ^f^	**!/X**	**√**	**√**
Diabetes mellitus^f^	**!/X**	**√**	**!/X**	**√**	**!/X**	**√**
Chronic kidney disease	**√**	**!**	**√**	**√**	**√**	**√**

**√√**: favored choice; **√**: may be favored choice; !: cautious use; = : may be acceptable depending on the clinical circumstances; X: relative contraindication;?: insufficient evidence to recommend for or against treatment. Therapies are presented in alphabetical order.

^a^ Typically used as combination therapy with IVGC/OGC (please check contraindications to IVGC/OGC therapy).

^b^ In patients with relapsed TED after OGC or IVGC treatment (cumulative dose 4.5 g), a second cycle of IVGC (cumulative dose <8.0 g) may be considered.

^c^ May be more efficacious in TED of relatively short duration (<9 months).

^d^ All Women of childbearing potential must use effective contraception during treatment.

^e^ Chronic hepatitis, tuberculosis.

^f^ Diabetic and hypertensive retinopathie*s* are contraindications to RT; uncontrolled diabetes is a contraindication to GC and TEP.

GC, glucocorticoid, RT, radiotherapy.

###### Summary of evidence

OGC and IVGC have been used and studied extensively in active moderate-to-severe TED.^74,82,83^ Available evidence shows efficacy for disease inactivation, marginal benefit on eye motility, and negligible benefit on proptosis. AEs are common from GC therapy, but overall, the safety profile is acceptable. The evidence also favors IVGC over OGC.

**Key Point 7.1.1.1:** IVGC therapy is a preferred treatment for active moderate-to-severe TED when disease activity is the prominent feature in the absence of either significant proptosis (see Section 2.1. for definition) or diplopia.

**Key Point 7.1.1.2:** Standard dosing with IVGC consists of IVMP at cumulative doses of 4.5 g over ∼3 months (0.5 g weekly × 6 weeks followed by 0.25 g weekly for an additional 6 weeks).

**Key Point 7.1.1.3:** Poor response to IVMP at 6 weeks should prompt consideration for treatment withdrawal and evaluation of other therapies. Clinicians should be alert for worsening diplopia or onset of DON that have occurred even while on IVMP therapy.

**Key Point 7.1.1.4**: A cumulative dose of IVMP >8.0 g should be avoided.

#### 7.1.2. Therapies for patients with moderate-to-severe TED unresponsive or intolerant to IVGCs

For patients who do not respond, partially respond, or are intolerant to IVGC therapy, RTX (see Section 7.1.4) and TCZ^84^ (see Section 7.1.6) may be considered. TEP (see section 7.1.3) has not been evaluated as salvage therapy in this setting. Other options, based on anecdotal evidence, are an additional course of IVGC (in patients with previous partial response, aiming not to exceed 8 g of methylprednisolone), or RT (see section 7.2). For patients whose disease is not progressive and who are not severely symptomatic, watchful monitoring is also an option.

**Key Point 7.1.2.1:** RTX and TCZ may be considered for TED inactivation in GC-resistant patients with active moderate-to-severe TED. TEP has not been evaluated in this setting.

#### 7.1.3. Teprotumumab

TEP is licensed only in the United States at the time of publication of this CS but is expected to be granted European Medicines Agency license in the future, hence its inclusion in this section.

##### Mode of action

A role of the IGF-1R in the pathogenesis of TED was suggested in early *in vitro* studies showing interactions between circulating TSH-R antibodies and the IGF-1R on orbital fibroblasts.^85,86^ Further evidence regarding the role of TSHR and IGF-1R crosstalk in the pathophysiology of TED emerged over the past decade.^87,88^

##### Clinical experience

TEP is the newest agent applied to the management of TED and paradoxically is the only drug approved by the Food and Drug Administration (FDA) for treatment of TED for patients ≥18 years of age, although methylprednisolone has long been FDA approved for “ocular inflammatory conditions unresponsive to topical corticosteroids.” More placebo-controlled trial data are available for TEP than for any other agent in current use, and it appears to be the most comprehensively effective therapy to date (see Efficacy and Table 5). Several important caveats need to be considered (see the Summary of Evidence section)

##### Efficacy

Early interest in the role of the IGF-1R in TED led to testing TEP, a fully human IGF-1R-inhibitory monoclonal antibody, in two placebo-controlled RCTs in patients with active moderate-to-severe disease.^89,90^

###### Composite outcome

In the first RCT comparing TEP with placebo, the primary outcome was defined as a composite of improvement in *both* CAS by ≥2 *and* reduction in proptosis by ≥2 mm at 24 weeks.^89^ This outcome was achieved by 69% (29/42) of patients assigned to TEP and 20% (9/45) of those receiving placebo. Among patients with baseline diplopia, there was improvement (defined as a minimum of one grade) in 68% (19/28) versus 29% (8/28) with placebo.

###### Activity

In the first RCT, the mean CAS score improved significantly more in the TEP-treated patients compared with placebo (3.4 vs. 1.85), and 69% of patients receiving TEP experienced disease inactivation to CAS of ≤1, compared with 21% of patients receiving placebo.^89^ In the second RCT (Treatment of Graves' Orbitopathy to Reduce Proptosis with Teprotumumab Infusions in a Randomized, Placebo-Controlled, Clinical Study [OPTIC]), disease inactivation (CAS ≤1) occurred by 24 weeks in 59% (24/41) of patients versus 21% (9/42) given placebo.^90^

###### Severity

In the first RCT, proptosis improved from baseline by a mean of 2.5 mm (vs. 0.15 improvement with placebo), and 40% (17/42) experienced proptosis reduction of ≥4 mm, compared with zero patients receiving placebo at 24 weeks.^89^ In the OPTIC trial, a proptosis reduction of ≥2 mm (the study's primary outcome) was achieved in 83% (34/41) of patients treated with TEP versus 10% (10/42) receiving placebo at 24 weeks, using an intention-to-treat analysis.^90^ Among patients with baseline diplopia in the OPTIC trial, there was improvement in 68% (19/28) versus 29% (8/28) with placebo.

A pooled analysis combining data on the 84 patients receiving TEP and 87 given placebo in the two RCTs showed a mean improvement in proptosis at 24 weeks of 3 mm in patients receiving TEP versus <0.5 mm in those given placebo. Diplopia improved in 70% (46/66) of patients treated with TEP versus 31% (18/59) of patients given placebo.^91^ A similar number of patients required additional medical or surgical treatments for TED with TEP (*n* = 8) and placebo (*n* = 11).^91^

###### Quality of life

In the first RCT, the visual functioning QOL improved significantly more with TEP than in the placebo group, whereas the appearance QOL subscale did not.^89^ In the OPTIC trial, the mean GO-QOL score improved by 13.8 points in TEP-treated patients versus 4.4 points with placebo, with significant improvement in both appearance and visual subscales.^90^ In the pooled analysis from these two RCTs, the visual function and appearance subscales both improved significantly more with TEP than with placebo (19.7 points vs. 7.0 points and 17.7 points vs. 5.6 points, respectively).^91^

###### Inactive disease and TED of longer duration

The response to TEP in patients with inactive (CAS ≤1) TED is currently being examined in an RCT (NCT04583735), with results expected in early 2023. A retrospective analysis of 31 patients with a mean TED duration of 81 months, with CAS ≤3 and without changes in diplopia or proptosis for >1 year, who received at least 3 infusions of TEP, found a mean proptosis reduction of 3.5 mm, and 90% (28/31) of patients experienced ≥2 mm reduction.^92^ Results from Treatment of Graves' Orbitopathy to Reduce Proptosis with Teprotumumab Infusions in an Open-Label Clinical Extension Study (OPTIX-X) provide additional data on the use of TEP in patients with TED of longer duration.^93^

Among 37 patients treated with placebo in OPTIC, who subsequently received TEP in OPTIC-X, the mean ± SD duration of disease was 12.3 ± 25 months, compared with 6.4 ± 2.4 months duration in OPTIC.^93^ Proptosis in these patients improved by ≥2 mm in 89% (33/37), diplopia improved in 61% (14 of 23), and CAS improved in 66% (21/32) of those with a baseline OPTIC-X CAS of >1.

##### Dosing and route of administration

TEP is given intravenously in eight doses, each 3 weeks apart. The first dose is 10 mg/kg, and the seven subsequent doses are 20 mg/kg.

##### Nonresponse and relapses after completion of treatment

Among the 34 patients showing a proptosis response of ≥2 mm in OPTIC, 10 patients (29.4%) experienced a relapse (described as “flare”) over the ensuing year, including 5 who had a proptosis relapse alone, 4 who experienced both a proptosis and CAS relapse, and 1 with a CAS relapse alone.^93^ Relapses had occurred at week 48 (27 weeks after final infusion) in seven patients, week 60 in two patients, and week 72 in one patient. The OPTIC-X study also examined the effect of a repeat course of eight TEP infusions in poor responders (*n* = 5) or those who relapsed after an initial study-defined response (*n* = 8) in OPTIC.

For the five nonresponders, two responded with proptosis reduction of ≥2 mm, one patient remained a nonresponder, and two dropped out due to either poor response or a serious adverse effect (intracerebral hemorrhage). For 8 patients among the 10 who relapsed after an initial response in OPTIC for whom data from OPTIC-X are available, 5 of 8 experienced proptosis reduction of ≥2 mm with the second course of TEP. An FDA briefing document cites a relapse rate of 37% at 72 weeks among TEP-treated patients (relapse defined as an increase in proptosis of ≥2 mm from week 24 in the study eye only) (https://www.fda.gov/media/133429/download).

###### Safety

TEP should not be used during pregnancy or for patients <18 years of age due to concerns regarding growth. The AE profile of TEP appears to be acceptable, but deterioration of glucose control in patients with diabetes or prediabetes, at times requiring insulin therapy, was noted in 10% of patients.^94^ Muscle cramps were reported in 25% of patients treated with TEP, nausea in 17%, alopecia in 13%, fatigue in 12%, and, importantly, hearing impairment in 10% of patients.^91^ A recent summary of five series reported hearing impairment in 29 of 190 (15.2%) patients treated with TEP, with resolution in 16 (55%) but persistence in 13 (45%) patients.^95^

Aggravation of inflammatory bowel disease (IBD) on TEP was noted in two patients in the two existing RCTs, and apparently new diagnoses of IBD have been described in conjunction with TEP therapy,^96^ so cautionary use of this drug is recommended in patients with this disorder. In the United States, the drug was granted FDA approval in 2020.

###### Cost

One course consisting of eight infusions of TEP has a retail cost of ∼$300,000, depending on patient weight, ∼2000 times that of IVGC.

###### Summary of evidence

The evidence for efficacy of TEP in patients with active moderate-to-severe TED of short duration with significant proptosis is convincing. However, 17–31% of patients treated did not meet the study definition for a response to treatment, 29–37% experienced disease relapse after an initial response,^91,94^ and data on improvement in nonresponders after a second course of TEP therapy are quite limited.^93^ Given lower costs and wider availability, IVGC may be preferred when the treatment target is purely inflammatory changes.

Further data related to TEP therapy, as with other therapies for TED, are needed in the following areas: (1) durability of improvement, (2) efficacy in inactive TED, (3) utility in patients unresponsive to initial therapy, (4) the ability to avoid subsequent medical therapy or rehabilitative surgeries, and (5) long-term safety. Additional trials to determine optimal dosing and duration of treatment, and direct comparisons are needed with other widely available therapies. These unknowns, as well as the high pricing, limited global availability, and absence of cost-effectiveness and comparative effectiveness data, prevent a complete appraisal of TEP's current role in the management of TED.

Cost-effectiveness appraisal is particularly important for TEP, given the high pricing of the drug in comparison with other treatments (Table 7). In the meantime, there is a case for all stakeholders, including professional organizations, insurers, health care providers, patients and their advocates, and drug manufacturers, to engage in discussions on how costly treatments for TED can be made more accessible. The manufacturer of TEP has recently developed a patient-directed cost assistance and insurance process online resource (https://www.tepezza.com/cost-and-support/).

**Key Point 7.1.3.1:** TEP is a preferred therapy, if available, in patients with active moderate-to-severe TED with significant proptosis (see Section 2.1. for definition) and/or diplopia.

#### 7.1.4. Rituximab

##### Mode of action

RTX targets CD 20 on activated B cells and impairs new antibody production and B cell-mediated helper function. It has been used extensively for lymphoma and some systemic autoimmune diseases.^97^

##### Clinical experience

RTX has been used for TED for the past 15 years. Approximately 160 patients have been reported in the literature to have received RTX for TED.^98,99^ There are only two small single-center RCTs with a total of 28 patients treated with RTX.^67,100^

##### Efficacy

###### Activity

The two RCTs are discordant with regard to the ability of RTX to induce inactivation compared with IVGC or placebo.^67,100^ The RCT demonstrating efficacy showed CAS decrease from baseline with both treatments (IVGC from 4.7 to 2.2, RTX from 4.4 to 0.6 at 24 weeks), and significantly greater CAS reductions after RTX (*n* = 15) than after IVGC (*n* = 16) at 16, 20, and 24 weeks.^67^ At 24 weeks, disease inactivation (CAS <3) occurred in significantly more RTX-treated patients than in IVMP-treated patients (100% vs. 69%).

The RTX group included patients (40%) who had been previously treated with steroids, but continued to have active moderate-to-severe TED. In the second RCT, RTX (*n* = 13) was compared with placebo (*n* = 12) and failed to demonstrate efficacy.^100^ Observational reports suggest efficacy.^98,99^

###### Severity

The RCTs and observational studies indicate little to no effect on proptosis (no different from placebo or IVGC in RCTs) or diplopia.

###### Quality of life

Modest improvements in GO-QOL were demonstrated by one of the RCTs^67^ and an observational study^101^ both from the same center, and the latter including some data from the former. In the RCT at 52 weeks follow-up, 77% (10/13) of RTX-treated patients reported improved eye functioning QOL and 62% (8/13) improved appearance, compared with rates of 54% (7/13) and 46% (6/13), respectively, with IVGC.^67^

##### Dosing and route of administration

Among the two RCTs, 64% (18/28) patients were treated with a total dose of 2000 mg RTX, the remainder with 500 mg RTX.^67,100^ A *post hoc* analysis of three studies from a single center has examined different dosing regimens of RTX in 40 patients and found equivalent rates of disease inactivation and absence of relapse with all doses of RTX.^102^

However, the 100 mg dose failed to lead to disease inactivation or prevent progression to DON in 14% (2/14) patients, and higher doses of RTX were associated with better diplopia outcomes, so the 500 mg was deemed to be optimal.^102^ Patients in the two RCTs^67,100^ were premedicated before receiving RTX using acetaminophen/paracetamol, intravenous hydrocortisone (100 mg) or IVMP (100 mg), and antihistamines.^67^

##### Nonresponse and relapses after completion of treatment

Nonresponse compared with placebo was reported in one small (*n* = 11) RCT.^100^ Among studies that have reported responses totaling ∼150 patients, relapses have not been reported.^101^

###### Safety

The rate of all AEs in the reported literature is 33–87%.^98^ Minor AEs ranged between 6% (for the 100 mg dose) and 75%.^53^ Serious AEs, mostly infusion reactions related to cytokine release with transient visual loss, but rarely fatal (described in patients with rheumatoid arthritis receiving long-term RTX 1000 mg every 6 months), are reported in 6–14% of cases. In a pooled analysis of the two RCTs, a total of 26 AEs occurred in 21 of 28 (75%) patients, including 1 case of vasculitis and 2 cases of transient vision loss due to cytokine release.^53^ Progressive multifocal leukoencephalopathy has been reported rarely in patients receiving RTX, generally for treatment of non-Hodgkin's lymphoma or other hematological malignancies.^103^

###### Cost

The cost of a 500 mg course of RTX is 24–28 times that of IVMP (Table 7).

###### Summary of evidence

There is contradictory evidence for the efficacy of RTX from two small single-center RCTs, but differences in baseline characteristics may explain the disparate results. Specifically, there was a shorter duration of TED in the study showing efficacy compared with the negative study (mean duration 4.5 months vs. 30 months).^53^ In addition, patients included in the negative study had higher CAS values, higher TRAb titers, and were more likely to be men and of older age, but less likely to be smokers than in the study showing benefit.^53^

The principal benefit is disease inactivation with no clinically significant effects on proptosis or diplopia. Modest effects on QOL have been reported in some reports. The response is durable at 1 year with a negligible relapse rate reported to date.^53^ Patients who have been previously treated with GCs and remain active with moderate-to-severe TED often respond to RTX.^101^ Doses between 100 and 2000 mg appear to be effective. On balance the evidence favors efficacy of RTX for disease inactivation (including previously GC-treated patients), with a low risk of relapse. Superiority to IVGC has been demonstrated in only one small RCT. The cost of RTX is significantly greater than that of IVMP.

**Key Point 7.1.4.1:** Evidence from RCTs is limited and divergent but suggests efficacy of RTX for inactivation of TED and prevention of relapses at >1 year, particularly in patients with TED of <9 months' duration.

**Key Point 7.1.4.2:** RTX therapy is acceptable in patients with active moderate-to-severe TED and prominent soft tissue involvement.

#### 7.1.5. Mycophenolate

##### Mode of action

Mycophenolate exerts its immunomodulatory effects by inhibiting guanosine monophosphate synthesis, T and B cell proliferation, suppresses antibody production, and interferes with chemotaxis.^104^

##### Clinical experience

Mycophenolate has been used in a large number of patients, mostly for prevention of transplant rejection, and in patients with autoimmune diseases.^105^ The published experience in TED is limited to two RCTs,^68,106^ one nonrandomized trial^107^ and one retrospective report.^108^

##### Efficacy

Two RCTs have studied mycophenolate in patients with active moderate-to-severe TED. The first RCT was a single-center study and compared GC with mycophenolate mofetil, both administered for 24 weeks.^106^ The second RCT compared IVGC given for 12 weeks with IVGC plus mycophenolate sodium for 24 weeks.^68^ A third study was a retrospective audit with a highly heterogeneous population of 20 patients with limited efficacy data and will not be considered any further.^108^ Finally, a recent nonrandomized trial examined the use of mycophenolate mofetil plus oral prednisolone in 242 patients with moderate-to-severe TED.^107^

###### Composite outcome

In the first RCT, the primary outcome was defined as improvement in ≥3 components of a composite, including improvement in CAS ≥2 or inactivation (CAS ≤3), improvement in soft tissue involvement by one grade in any of the following: eyelid swelling, eyelid erythema, conjunctival redness or conjunctival edema, reduction in proptosis ≥2 mm, improvement in eye movement (disappearance or reduction in severity of decreased eye movements), improvement in diplopia, or an increase in visual acuity ≥2/10.^106^ The primary outcome favored mycophenolate at 12 weeks, with 79% achieving the primary outcome versus 51% of those given IVGC, and at 24 weeks (91% vs. 68%).

The second RCT, which used the EUGOGO composite index (improvement defined as greater than or equal to two components among eyelid swelling, CAS, proptosis, lid width, diplopia, or eye muscle motility),^19^ found no differences in the composite index at 12 weeks between IVGC and IVGC plus mycophenolate sodium, and no differences in relapse rates at 24 and 36 weeks in the two groups.^68^ However, in a *post hoc* analysis, a significantly greater improvement was detected in mycophenolate-treated patients at 36 weeks, with 67% (49/73) of patients improving vs. 46% (31/68) patients improving with IVMP.^68^

###### Activity

The first RCT found significant reductions in CAS within each group from baseline.^106^ Comparisons between groups showed no difference in mean CAS at 12 or 24 weeks, but the proportion of patients with disease inactivation, defined as CAS ≤3/10 at 24 weeks, favored mycophenolate mofetil, with inactivation occurring in 94% (69/80) of patients treated with this drug versus 69% (54/78) of those treated with GC. In the second RCT, CAS improved from baseline in both groups but there were no differences between groups.^68^

###### Severity

The first RCT found a similar degree of improvement in proptosis in both groups at 24 weeks (mycophenolate mofetil −3.4 mm vs. GC −2.2 mm), but improvement occurred in a significantly higher percentage of mycophenolate mofetil-treated patients (69%, 55/80) than in those receiving GC (40%, 31/78).^106^ Diplopia improved in both groups at 24 weeks and the response was significantly better with mycophenolate than with GC (90%, 47/52 vs. 64%, 35/55). DON was not reported during follow-up in either group.

In the second RCT, proptosis and diplopia did not change from baseline in either group and there was no difference between groups, while DON occurred in both groups, including 9% (7/75) of patients receiving mycophenolate plus IVMP and 6% (4/72) of patients receiving IVMP alone.^68^ The nonrandomized study of 242 patients with moderate-to-severe TED noted improvement in proptosis, and diplopia in 83% and 94.2% of patients, respectively, at 12 months.^107^

###### Quality of life

QOL was not assessed in the first RCT.^106^ In the second RCT, patients in both arms of the study noted slight improvement in QOL (<10 points improvement on the GO-QOL questionnaire), but there was no difference between groups.^68^

##### Dosing and route of administration

The first RCT compared GC in the form of IVMP 0.5 g on 3 consecutive days for 2 consecutive weeks followed by oral prednisone 60 mg daily for 8 weeks, and then tapering over the final 14 weeks (giving a cumulative dose of 6.7 g dose, for 24 weeks), with mycophenolate mofetil 500 mg twice daily for 24 weeks.^106^ The second RCT used IVGC 4.5 g cumulative dose for 12 weeks compared with IVGC (same regimen) plus mycophenolate sodium 360 mg twice daily for 24 weeks.^68^

##### Nonresponse and relapses after completion of treatment

The first RCT reported “reactivation” (without providing a definition) in 6.4% (5/78) GCs versus 0% (0/80) in the mycophenolate mofetil group.^106^ In the second RCT, relapses occurred in both groups, but between-group differences were not significant at 24 weeks [combination therapy 8% (4/53), IVGC monotherapy 11% (4/38)] or at 36 weeks [8.2% (6/73) versus IVGC 10.3% (7/68)].^68^

###### Safety

In the first RCT, the rate of all AEs was significantly higher with GC, occurring in 28% (22/79) compared with mycophenolate mofetil occurring in 5% (4/80).^106^ The serious AE rate was 1.3% (1/79) for GC and 0% (0/80) for mycophenolate mofetil. In the second RCT, mild and moderate (grade 1–2) AEs also occurred in both groups including 47% (39/83) of patients treated with IVGC plus mycophenolate mofetil versus 36% (29/81) for those receiving GC alone, without statistically significant between-group differences. Serious AEs also occurred to a similar extent in both groups, including 16% (13/83) of patients receiving mycophenolate mofetil plus IVGC and 12% (10/81) of those given IVMP alone.^68^

###### Cost

The cost of a course of mycophenolate mofetil as described in the two RCTs is between five and seven times that of a course of IVGC^68,106^ (Table 7).

###### Summary of evidence

The first RCT demonstrated significant superiority of mycophenolate mofetil compared with IVGC in primary end points (composite outcome), as well as CAS, proptosis, diplopia, relapses, development of DON, and safety.^106^ Indeed, the response to mycophenolate mofetil in this population far exceeded that reported for any medical treatment for TED. Conversely, the second RCT was negative in terms of its primary objectives, although a significant difference in the composite outcome at 36 weeks (but not at 12 or 24 weeks) was observed in a *post hoc* analysis, of uncertain clinical significance.

Although there were differences between the two study populations within demographics such as age and geographical location, smoking history, the use of concurrent therapy, and actual dose and preparation of mycophenolate delivered, these disparate outcomes are not easily explicable, and the lack of additional data to help understand the discrepancies suggests a need for additional efficacy data, to better define the role of this drug in TED. Recently, combination therapy IVMP plus mycophenolate was recommended as first-line therapy for TED in the EUGOGO clinical practice guidelines,^109^ but the limited data and inconsistent findings to date were deemed by the TF to be not sufficiently convincing.

#### 7.1.6. Tocilizumab

##### Mode of action

Interleukin-6 is expressed in orbital fibroblasts of patients with TED and seems to drive inflammation.^2^ TCZ is an interleukin-6 receptor blocker.

##### Clinical experience

TCZ has been used extensively for inflammatory arthritis.^110^ Reports on its use in TED in the published literature are confined to <100 patients mostly from a single center.^84,111,112^

##### Efficacy

###### Activity

TCZ was shown to be effective in inactivating TED in all treated patients of a small open-label study involving 18 patients with CAS ≥4.^111^ A small RCT followed in 32 GC-resistant patients with moderate-to-severe TED and baseline CAS ≥4.^112^ The primary end point (improvement in CAS by ≥2 at 16 weeks) was achieved in significantly more treated patients than those receiving placebo (93%, 14/15 vs. 59%, 10/17); however, there was no difference between groups by week 40. A real-world report of 54 patient with GC-resistant TED treated with TCZ for 9 years from the same center as the original open label study cited inactivation in 74% of patients.^84^

###### Severity

The open-label study showed reduction in proptosis by a mean 3.92 mm in patients with GC-resistant TED and resolution of diplopia in 54% (7/13) of patients.^111^ The real-world study reported proptosis reduction ≥2 mm from baseline in 78% (42/54) of patients, and improvement in diplopia in 68% (19/28) of patients.^84^). In the RCT, proptosis values in TCZ-treated patients were significantly lower than those in placebo-treated patients at 16 weeks by a median of 1.5 mm; however, no differences in proptosis were demonstrable at 40 weeks, and diplopia improved in only 7% (1/15) of patients treated with TCZ.

Despite modest improvement in individual parameters, an objective composite index improved significantly more in TCZ- than in placebo-treated patients at 16 weeks (73%, 11/15 vs. 29%, 5/17) and this was sustained at 40 weeks (67%, 10/15 vs. 18%, 3/17).^112^

###### Quality of life

In the RCT, QOL (GO-QOL and SF-36) improved more in the TCZ group than in the placebo group at 16 weeks, but there were no differences at 40 weeks.^112^ The observational studies^84,111^ did not report on QOL.

##### Dosing and route of administration

The studies in TED patients have used intravenous TCZ 8 mg/kg or placebo on weeks 0, 4, 8, and 12.^84,111,112^ A subcutaneous preparation of TCZ is now available and requires further exploration in TED.^113,114^

##### Nonresponse and relapses after completion of treatment

In the RCT, the nonresponse rate based on the primary end point (improvement in CAS by ≥2) compared with baseline was 7% (1/15) at 16 weeks and 13% (2/15) at 40 weeks.^112^ The RCT did not include relapses in its analysis.^112^ Relapses were not observed in the open-label study.^111^ The real-world study reported relapses in 7.4% of patients.^84^

###### Safety

AEs include risk of severe infections, hepatotoxicity, and anaphylaxis. The RCT reported a total of 58 AEs in the TCZ and 33 in the placebo-treated patients by 40 weeks and included 2 serious AEs (transaminase elevation, pyelonephritis) among the 15 TCZ-treated patients.^112^ The observational studies^84,111^ reported mild or moderate AEs such as fatigue, upper respiratory infection, cellulitis, neutropenia, and mild transaminase elevation, occurring in up to 48% of patients.^84^

###### Cost

The cost of a course of TCZ is 60–85 times that of IVGC (Table 7).

###### Summary of evidence

The impressive outcomes from the observational studies (especially on proptosis)^84,111^ have not been reproduced to the same degree by a single small RCT, although overall efficacy of TCZ was confirmed in GC-resistant patients with TED.^112^ An ongoing multicenter trial is testing intravenous TCZ efficacy in comparison with IVGCs and will further inform on the place of this drug in the routine management of TED (EudraCT Number: 2018-002790-22, ClinicalTrials.gov Identifier: NCT04876534).

**Key Point 7.1.6.1:** TCZ is an acceptable treatment for TED inactivation in GC-resistant patients with active moderate-to-severe disease.

#### 7.1.7. Other agents

##### 7.1.7.1. Other agents tested in TED patients and clinically available

Several additional agents have been tried in TED (e.g., atorvastatin, methotrexate, intravenous immunoglobulin (IVIG), azathioprine, cyclosporine, somatostatin analogues, and tumor necrosis factor (TNF) alpha inhibitors. Only a few have been studied in RCTs.

An RCT comparing atorvastatin 20 mg daily × 24 weeks plus IVMP (500 mg IV weekly × 6 weeks followed by 250 mg weekly × 6 weeks) with IVMP alone found significantly greater improvement in the EUGOGO composite index (51%, 21/41, vs. 28%, 11/39 patients), and relapses at 24 weeks were less likely in the atorvastatin plus IVMP arm (0/41 patients) versus the IVMP alone arm (15%, 6/39 patients).^71^ The GO-QOL improved significantly more in the combined therapy group (by 6.4 points) compared with that in the IVMP group.

Despite greater improvement in the composite index when atorvastatin was added to IVMP, there were no significant differences between groups in individual eye components such as CAS and diplopia, which improved in both groups, or proptosis, visual acuity, and eye aperture, which improved in neither group.^71^

IVIG appeared to have efficacy comparable with OGC in the one and only RCT,^115^ but because of the high cost, risk of transmission of infections and availability of other treatments, IVIG is not currently used in TED.

The roles of azathioprine (one RCT), cyclosporine (two RCTs) TNF alpha inhibitors, somatostatin analogues (four RCTs), and methotrexate are questionable as the evidence is either anecdotal or indicates lack of efficacy, or the side effect profile is unfavorable.^65^ Unfortunately, the evaluation of these agents has been done utilizing a multitude of outcomes along with different definitions for relapse rates after a successful outcome, thus precluding an easy comparison between these agents.

##### 7.1.7.2. Other agents under investigation in TED patients but not clinically available

A recent study aimed at decreasing the half-life of IgG with a neonatal fragment crystallizable receptor inhibitor (IMVT-1401) was terminated early due to concerns about dyslipidemia (ClinicalTrials.gov Identifier: NCT03938545). Belimumab, an anti-B cell activating factor monoclonal antibody, was compared with IVGCs in a randomized trial (EudraCT Number: 2015-002127-26)^116^ with potentially promising results that have not been published at the time of this writing.

##### 7.1.7.3. Other agents tested in GD patients with potential benefit in TED but not clinically available

Inferentially, a group of agents that have been tested as therapy for GD could ultimately prove beneficial for TED. Iscalimab blocks TSHR activation through the inhibition of intracellular activities leading to TRAb formation,^117^ and ATX-GD59 is intended to induce tolerance to TSHR.^118^ Both agents have been tested in small studies with encouraging results. A TSHR blocking monoclonal antibody (K1-70) (ClinicalTrials.gov Identifier: NCT02904330) is showing encouraging results in GD and also improvement in TED in the few patients studied who had both conditions.^119^

This was a phase 1 study and further investigation of this therapy is needed before a clear indication for TED can emerge. This and other planned studies with small molecule antagonists to the TSHR (S37a, ANTAG3) will possibly add to the armamentarium against TED in the future.

### 7.2. Radiotherapy for moderate-to-severe TED

RT has been used to treat TED for >70 years and may work by inhibiting or depleting lymphocytes and fibrocytes in the involved orbital tissue. The efficacy of RT for TED is variable in clinical studies to date, and interpretation is hampered by divergent inclusion criteria and outcome analyses.^120^ Proponents of RT cite a reduction in periocular inflammation in 60% of patients with active TED, a rate equivalent to OGC but less than that seen with IVGC.^121^ Data from two observational studies have shown a prolonged duration of effect from RT that may provide a GC-sparing effect, allowing an earlier tapering of OGC.^122,123^

RT has been compared with sham RT in three prospective studies. Two trials from the Netherlands randomized a total of 147 subjects with progressive TED and found the irradiated group ultimately had better ocular motility, manifested by improved excursions and less diplopia.^124,125^ Conversely, an American RCT comparing RT on one eye with sham therapy on the opposite side in 42 subjects with longer standing disease (median TED duration 1.3 years, range 0.2–16 years) found no benefit in a composite outcome of proptosis, lid retraction, and soft tissue index.^126^ The latter study supports the observation that RT is ineffective for late-stage or inactive disease.

Several studies have assessed the benefit of adding RT to GC therapy in TED. Two small RCTs with a combined total of 40 participants with active TED found greater response based on global severity scores in the combined RT plus OGC group than in the OGC control group.^127,128^ A retrospective Canadian study reviewed 351 patients with progressive TED who received either IVGC alone or IVGC combined with RT. At an average of 3.2 years follow-up, DON had developed in 17% of the IVGC group but in none of the combined therapy group, and the group with adjunctive RT also had a significantly greater improvement in ocular motility.^77^

Two additional retrospective analyses comparing IVGC with or without RT noted marginally increased benefit in the combined therapy group.^129,130^ However, a recent RCT from the United Kingdom (CIRTED Trial) found no gain from the addition of RT to OGC in subjects with active TED and moderately severe disease, in terms of a binary composite clinical outcome score or in terms of CAS.^64^ It is unclear whether the addition of oral or IVGCs amplifies the clinical response to RT.

The standard dosing protocol for early progressive disease since 1973 is 20 Gray (2000 Rads) divided over 10 days, delivered to the retrobulbar orbit through a lateral port, avoiding ocular or intracranial exposure.^120^ Two studies found equivalent efficacy when doses were reduced or divided into a greater number of fractions.^131,132^

Modern linear accelerator RT units have an improved safety record with retrospective series in TED showing no increased risk of cataracts,^133^ although a benign meningioma in the radiation field has been identified in a case report.^134^ Because of a theoretical lifetime risk of developing tumors, its use for TED is relatively contraindicated in people <35 years. RT may also increase the incidence of retinal vascular disease in patients with diabetes mellitus or hypertension.^120^ Orbital edema may increase during RT but can be controlled by concurrent GC.

**Key Point 7.2.1:** RT is a preferred treatment in patients with active moderate-to-severe TED whose principal feature is progressive diplopia.

**Key Point 7.2.2:** RT should be used cautiously in diabetic patients to avoid possible retinopathy. It is relatively contraindicated for those younger than 35 years of age to avoid a theoretical lifetime risk of tumors developing in the radiation field.

### 7.3. Surgical intervention for inactive moderate-to-severe TED

#### 7.3.1. Surgical intervention overview

Elective surgery to correct proptosis, strabismus, eyelid malposition, and fat pockets can be initiated in inactive TED where clinical stability has been maintained and a euthyroid status achieved before surgery. Ocular motility should generally be stable for 4–6 months before strabismus surgery is performed. Surgical rehabilitation for TED is a staged approach, addressing proptosis first, then strabismus, and eyelid changes last. Not all patients require all procedures. QOL improvements often occur as a result of surgical rehabilitation for TED.^135,136^

**Key Point 7.3.1.1**: Surgery for moderate-to-severe TED should be performed by an orbital surgeon experienced with these procedures and their complications.

**Key Point 7.3.1.2**: Rehabilitative surgery for moderate-to-severe TED should only be performed when the disease is inactive and euthyroidism has been achieved and maintained.

#### 7.3.2. Orbital decompression

Orbital decompression reduces intraorbital pressure and proptosis resulting from expanded orbital tissues by removal of bony walls, resection of orbital fat, or both. Indications include disfiguring proptosis, chronic orbital congestion, globe subluxation (Fig. 2f), and DON. The outcomes and complications for DON decompression surgery are covered in Section 8.3.

The most common indications are to restore appearance in proptosis and improve comfort in congestive orbitopathy and exposure keratopathy. In mild cases, intraconal orbital fat may be resected in fat-predominant disease, or the lateral wall drilled or partially removed. Greater reduction may be achieved by removing the bony medial wall and/or floor, opening the periorbital envelope, and displacing orbital fat and muscle into adjoining sinuses. Approximately 2 mm of proptosis reduction may be expected for each wall removed or 2 cm^3^ of fat excision.^137,138^

A rare indication is to relieve longstanding soft tissue congestion. Affected individuals have high CAS/VISA inflammatory scores but have had no recent progression and are nonresponsive to medical intervention. Improved venous drainage after expansion of the orbital compartment can result in dramatic improvement in orbital soft tissue changes and relieve orbital pain (Supplementary Fig. S2a, b).

Specific complications are associated with each wall decompressed. Deep lateral or medial wall surgery may cause a cerebrospinal fluid leak from dural injury,^139^ while oscillopsia (visual bobbing) may result from adhesions between the lateral rectus and temporalis muscles. Cheek numbness and inferior displacement of the globe may occur with floor decompression, while sinusitis and anesthesia of the upper jaw and nose may result from medial wall surgery. New-onset strabismus may develop in 7–34% of cases, depending on factors such as the technique of orbital decompression used and the size and restriction of enlarged extraocular muscle.^140^

This is less common in cases of fat-targeted disease, with one large series showing new diplopia persisting at 6 months after retro-orbital fat dissection in 8.6% of patients.^141^ A smaller fat-to-orbit ratio is associated with a lower likelihood of developing new diplopia postoperatively.^142^

**Key Point 7.3.2.1:** The specific surgical approach should be tailored to the indication (DON, proptosis), type of orbitopathy (muscle or fat predominant congestive disease), and desired reduction in proptosis.

#### 7.3.3. Strabismus procedures

Strabismus with diplopia and/or a compensatory head turn to restore monocular gaze may develop from initial swelling and subsequent fibrosis of affected EOMs, or complicating orbital decompression surgery. While waiting for the diplopia to stabilize, binocular single vision in the primary or reading position may be obtained by using Fresnel adhesive prisms applied to a spectacle lens. In cases where prismatic correction is ineffective, diplopia can be avoided by occluding the worst affected eye with a foil, tape, or contact paper on the spectacle lens. Injection in the affected muscle with botulinum toxin is occasionally used as a temporary measure to correct diplopia.^143^

The goal of strabismus surgery is to restore or expand the field of binocular single vision (Supplementary Fig. S2e, f) and hence improve QOL.^144^ Once strabismus measurements have stabilized for at least 6 months, the restricted rectus muscles are typically recessed by releasing them from their insertion site and reinserting them by a variable amount further back in the globe, based on the desired correction, through a transconjunctival approach. Adjustable sutures may be used, which can be shifted after wakening the patients based on their feedback.^145^ Muscle tendons may be lengthened using donor tissue or hang-back sutures for large deviations.^146^

In severe strabismus, several surgical procedures on different muscles may be required and the field of binocular single vision may remain limited. After a large inferior rectus muscle recession, secondary lower lid retraction may develop. Patients deferring surgery or with smaller deviations may be helped with permanent prisms ground into the spectacle lenses.

**Key Point 7.3.3.2:** In patients with diplopia and inactive TED, binocular single vision in the primary position of gaze may be restored with strabismus surgery or permanent prisms ground into the spectacle lenses.

#### 7.3.4. Eyelid procedures

Eyelid correction is performed in stages, usually addressing upper or lower retraction first, and concerns about appearance such as swelling or the adjacent glabellar folds second (Supplementary Fig. S2 g, h and i, j). In cases with significant proptosis, a preceding decompression surgery often results in a better reconstructive outcome from the lid surgery. Upper lid retraction may result from a fibrotic levator muscle or in compensation for a restricted inferior rectus muscle and is characterized by scleral show, lateral flare (retraction) (Fig. 2d), and lagophthalmos (Fig. 2a).

During the early progressive phase, upper lid retraction may temporarily respond to triamcinolone injection into the supratarsal subconjunctival space.^147^ The upper lid may be lowered by releasing the retractor muscle from an anterior or posterior approach.^148^ The retracted lower lid may be elevated with the use of autologous or allograft spacer materials.

Correction of upper lid fat prolapse in TED is achieved with a customized blepharoplasty addressing the excess of the preaponeurotic and sub-brow fat pads, and lacrimal gland prolapse. Botulinum toxin can be injected into the muscles between the brows to relax the vertical frown line.

**Key Point 7.3.4.1**: Eyelid retraction and fat prolapse are surgically corrected when TED is inactive and euthyroidism is achieved, and after decompression and strabismus surgery as indicated.

## 8. THERAPY FOR SIGHT-THREATENING TED

### 8.1. Intravenous glucocorticoids

DON may result from compression of the optic nerve by enlarged EOM at the apex of the orbit (Fig. 3a, b), or infrequently (<5%), due to stretch of the nerve because of proptosis. It is important to distinguish these two forms radiographically, as optic nerve stretch does not respond to medical treatments and requires surgical decompression to reduce proptosis.^24^

For many years, orbital decompression has been the standard treatment for DON but IVGCs have proven effective as well, and are now used first, to possibly avoid surgery.^149^ Although the optimal dose and schedule of GC are not established, the recommended use of large doses (0.5–1.0 g) of IVMP daily for 3 consecutive^150^ or alternate days,^151^ is based on the experience of treating patients with optic neuritis from other etiologies.^152^

The existing literature defines the response to IVGC rather broadly as “visual recovery,” but does not provide quantitative data on improvements in visual fields and color vision. IVGC has been reported to be effective in ∼40% of DON patients, generating improvements in visual acuity and avoiding subsequent orbital decompression.^151,153^ Therefore, IVGC should generally be considered as the preferred treatment with the purpose of avoiding or postponing surgery.^151^ The presence of optic disk swelling or atrophy at diagnosis are predictors of inadequate response to IVGC,^153^ but should not deter a trial of these drugs to assess efficacy in a particular patient.

Visual deterioration 2 weeks after initiating therapy is also predictive or poor response to IVGC. Although late surgical decompression can still provide benefit for DON, it may not allow complete restoration of normal visual function.^154–156^ Recent reports of effectiveness at treating DON by mycophenolate,^108^ TEP,^157^ and TCZ^158^ require confirmation in RCTs.

**Key Point 8.1.1:** Patients with DON require urgent treatment with IVGC therapy, with close monitoring of response and early (after 2 weeks) consideration for decompression surgery if baseline visual function is not restored and maintained with medical therapy.

### 8.2. Radiotherapy in DON

The role of combined RT and GC in prevention of DON in high-risk patients and in reducing the need for surgical decompression in patients with existing DON remains controversial. Evidence from three large retrospective studies indicates that this approach may reduce the incidence of DON in high-risk patients^77^ and may delay or obviate the need for decompression surgery in patients with established DON.^159,160^ A prospective study is currently underway by the International Thyroid Eye Disease Society (ITEDS) to confirm this preventive application (Clinical Trials.gov identifier: NCT02339142).

Most patients with DON or at high risk of DON (Table 2) have progressive diplopia or reduced ocular motility and so are already candidates for RT (Section 7.2, Key Point 5.2.1) and likely to benefit from such treatment.

**Key Point 8.2.1:** RT may be considered for preventing or as an adjunct to treating DON.

### 8.3. Orbital decompression for DON

Orbital decompression has been recommended for cases of recent-onset or progressive DON who respond incompletely or only transiently to immunosuppressive therapy.^151^ In most cases, apical compression of the optic nerve by swollen EOMs is relieved by decompression of the deep medial and inferior orbital wall through a transcaruncular or transnasal endoscopic approach. Visual improvement may be noted within days of the procedure, and even severe or longstanding visual loss may have partial or full visual recovery.^24^

Strabismus is more likely from these surgeries as the muscles are already inflamed.^24^ Complications include cerebrospinal fluid rhinorrhea or rarely an intracranial hemorrhage.^161^ Orbital decompression for the rare case of stretch optic neuropathy is usually designed to maximize reduction of proptosis by expansion into adjoining sinuses and fat excision.^162^ Patients who require orbital decompression for DON during the active progressive phase of TED may require adjunctive therapy with medical treatments or RT aiming to inactivate the disease (Table 5).

Occasionally vision loss may persist due to irreversible optic nerve atrophy despite combined medical and surgical therapy.^163^ Risk factors include advanced age, comorbidities such as diabetes mellitus, and delays to treatment. Poor response to a trial of IVGC and evidence of optic nerve atrophy on OCT predict a less favorable outcome. A postoperative CT scan can indicate whether additional surgical apical decompression is possible.

**Key Point 8.3.1:** In patients with compressive DON, orbital decompression of the deep medial wall and orbital floor should be considered to restore vision by reducing apical compression on the optic nerve.

## 9. OVERVIEW OF THE MANAGEMENT OF TED

Figure 5 shows an overview of the suggested management of TED. Despite great progress in recent decades, the management of TED remains a challenge (except in the mildest cases). Because of clinical disease heterogeneity and insufficient published evidence on this topic (i.e., scarcity of rigorous RCTs), robust recommendations regarding first-line and second-line treatments are challenging. An individualized approach to the management of TED, based on disease activity, severity, duration, trend across time, impact of the disease on daily living, treatment goals, patient age, and comorbidities, as well as the availability and relative costs of such therapies, is advised.

Treatment options during both the active phase (generally, immunomodulatory drugs) and the inactive phase (generally, corrective surgical procedures) should be carefully discussed with patients. Finally, regional and even local health care system differences impact the availability of current therapies, and these factors become critical in the individualization of care.

## 10. RESEARCH GAPS IN THE MANAGEMENT OF TED

Table 9 lists gaps in the understanding of TED and its management that the TF deemed to have importance as the focus of further clinical research.

**Table 9.** Research Gaps in the Management of Thyroid Eye Disease

Identifying TED or those at risk for TED
Are there reliable biomarkers to predict the development of TED in patients with newly diagnosed GD?
Are there reliable biomarkers to assess TED activity more accurately than CAS?
Is there a simple clinical screening tool to identify patients with early TED?
Is there a simple and easy screening tool that patients with GD can use to self-diagnose TED early?
Is race a risk factor for TED?
What are the underlying mechanisms whereby radioactive iodine increases the risk of TED?
Assessment of patients with TED
How does vision, inflammation, strabismus, appearance compare with CAS for reproducibility and for predicting response to treatment?
Are there more objective and reproducible methods than clinical examination to document the features of TED (e.g., photogrammetry)?
How do we best utilize QOL measures (e.g., GO-QOL, TED QOL) to guide everyday clinical practice?
Treatment of mild TED
Is selenium useful in selenium sufficient areas?
Is elevation of head of bed of any value in patients with TED?
Treatment of moderate-to-severe TED
How does TEP compare with IVGC therapy in head-to-head comparison studies?
What is the durability of clinical response after TEP therapy?
What is the optimal dosing and duration of TEP therapy?
Is TEP therapy cost-effective at current prices?
What is the effectiveness of TEP therapy for inactive and/or protracted TED (>12 months duration)
What is the role of mycophenolate mofetil?
Is there a role for thyrotropin receptor blocking agents in the management of TED?
Is combined treatment of IVGC and RT more efficacious than IVGC alone?
What is the efficacy and optimal dosing of RTX?
What are the most relevant outcome measures in clinical trials for TED?
What is the impact of medical therapies on subsequent surgical management?
Is selenium helpful in moderate-to-mild TED?
Is there a role for statins?
Treatment of recurrent or refractory TED
What are the most effective treatment choices for recurrent TED?
Pathogenesis of TED
What components of tobacco smoke contribute to TED?
How effective is smoking cessation?
What is and how do we separate “congestive” TED from active TED?
Health care models for the management of TED
What is the most clinically effective and cost-effective specialty TED care model?
What is the impact of current drug costs, affordability, and limited global availability on health disparities in TED?
Ophthalmology-specific research
What is the role of chin-up positioned eye assessment in TED (to eliminate gaze-dependent ocular hypertension and optic neuropathy in restrictive strabismus)?
Is the Gorman diplopia score an optimal metric of ocular motility impairment in routine clinical practice and in clinical trials?
What is the role of RT/GC vs. GC alone in treating cases of established DON and allowing avoidance of surgery?

GD, Graves' disease; QOL, quality of life.

## Supplementary Material

Supplemental data

Supplemental data

Supplemental data

Supplemental data

## DISCLAIMER

The views expressed in this article are those of the authors and do not reflect the official policy of the Department of Defense, the National Institutes of Health, or the U.S. Government. Drs. Burch and Leung are employees of the U.S. Government. This study was prepared as part of their official duties. Title 17U.S.C. 105 provides the “Copyright protection under this title is not available for any work of the U.S. Government.” Title 17U.S.C. 101 defines a U.S. Government work as a work prepared by a military service member or employee of the U.S. Government as part of that person's official duties.

## ENDORSEMENTS

The final document was officially endorsed by the Latin American Thyroid Society (LATS) and Asia and Oceania Thyroid Association (AOTA).
