# Supplementary material for: Management of Thyroid Eye Disease: A Consensus Statement by the American Thyroid Association and the European Thyroid Association
Source: Thyroid. 2022 Dec 13;32(12):1439–70. doi: 10.1089/thy.2022.0251 (PMC9807259; doi:10.1089/thy.2022.0251)
Supplement: Supplemental data [file Supp_FigS3.docx]

**Figure S3: Diagnostic criteria and suggested office-based examination by endocrinologists for assessment of TED**

**
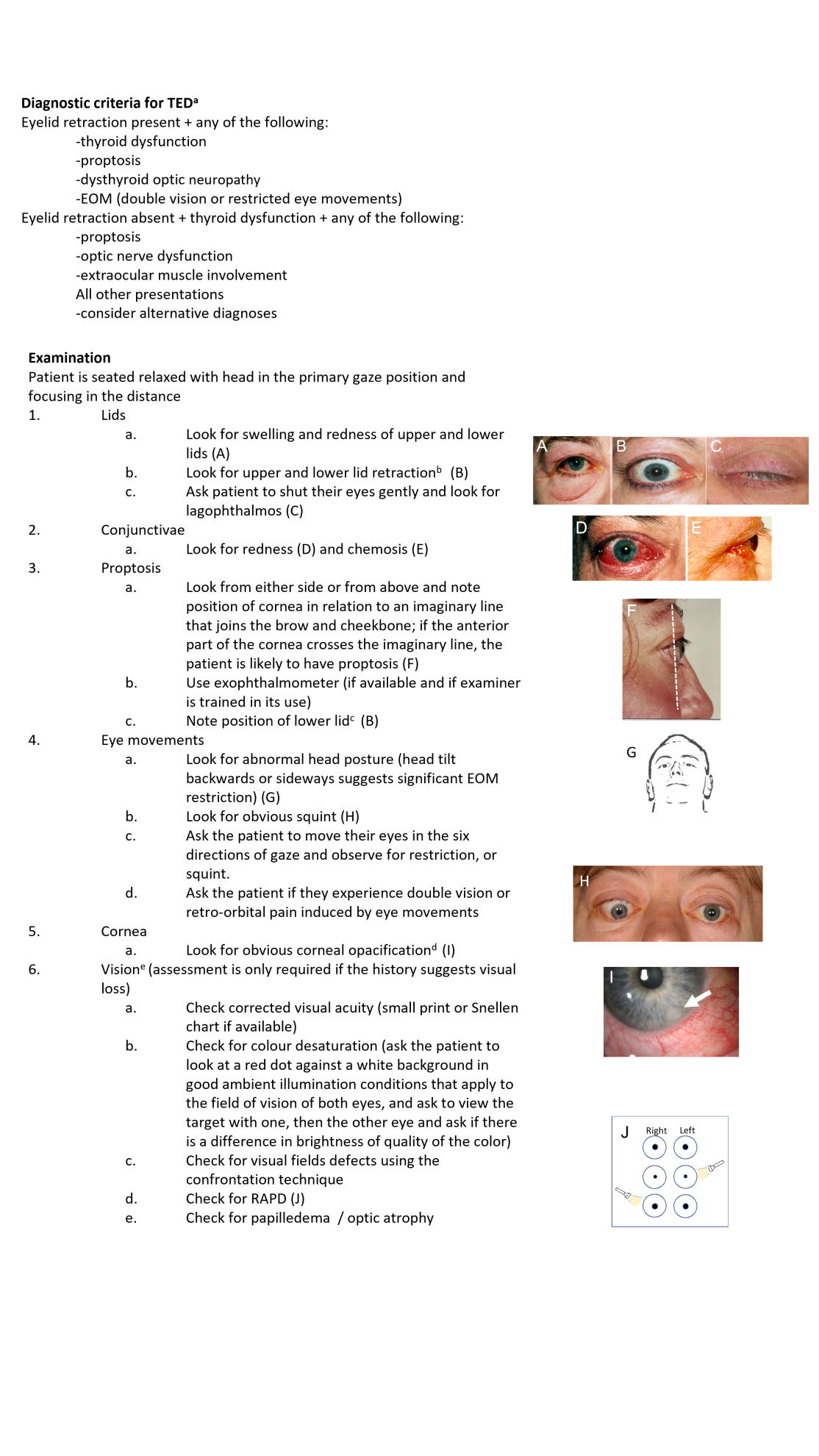
**

**Footnotes Figure S3:**

^a^From Bartley GB, Gorman CA. Diagnostic criteria for Graves' ophthalmopathy. Am J Ophthalmol. 1995 Jun;119(6):792-5.

^b^Upper lid retraction may be a manifestation of thyrotoxicosis

^c^There is a correlation between lower lid retraction and proptosis and if present is collateral evidence for proptosis

^d^Very rarely seen in an endocrine clinic

^e^Dysthyroid optic neuropathy is rare and an unlikely cause for visual problems in the absence of significant EOM restriction or double vision

Images A, and D with permission from publisher taken from Dickinson AJ, Perros P. Controversies in the clinical evaluation of active thyroid-associated orbitopathy: use of a detailed protocol with comparative photographs for objective assessment. Clin Endocrinol (Oxf). 2001 Sep;55(3):283-303. Images E, F, G and I with patients consent (courtesy of P Perros), B, C and H with patient consent (courtesy of P Dolman)

**Figure S3 Abbreviations:** DON: dysthyroid optic neuropathy; EOM: extraocular muscle; RAPD: relative afferent pupillary defect
