# Supplementary material for: Management of Thyroid Eye Disease: A Consensus Statement by the American Thyroid Association and the European Thyroid Association
Source: Thyroid. 2022 Dec 13;32(12):1439–70. doi: 10.1089/thy.2022.0251 (PMC9807259; doi:10.1089/thy.2022.0251)
Supplement: Supplemental data [file Supp_FigS1.docx]

**Supplementary Legends to Figures (S1, S2, S3)**

**Figure S1: Objective eye measurement in TED**

**a, b**: Estimating ocular ductions with corneal light reflex: The examiner shines a penlight on the patient’s eyes and observes the light reflex on the cornea as the patient is instructed to look in 4 directions, up, down, right, and left (Courtesy of P Dolman).

**a**. Light reflexes on cornea (white arrows) as patient attempts upgaze. Right light reflex is midway between limbus and pupil edge, so eye is elevating 30^o^. Left eye reflex is inside pupil and upgaze limited to 10^o^

**b**. Right eye looking up: Light reflects from the limbal edge meaning the patient has full duction of 45^o^. If upgaze is limited so that the light reflects from the pupil edge, the patient duction is restricted to 15^o^ and if it is seen midway between the pupil edge and limbus, the duction is restricted to 30^o^.

**c.** Exophthalmometer allowing protrusion of eye to be measured through the prism (Courtesy P Dolman).

**d.** Swinging light test: Patient with right afferent pupillary defect. Penlight shone on left normal eye elicits a full bilateral pupillary constriction. Penlight shone on right eye with optic nerve conduction deficit elicits a lesser pupillary constriction, causing apparent relative dilatation of both pupils (courtesy of H Burch).


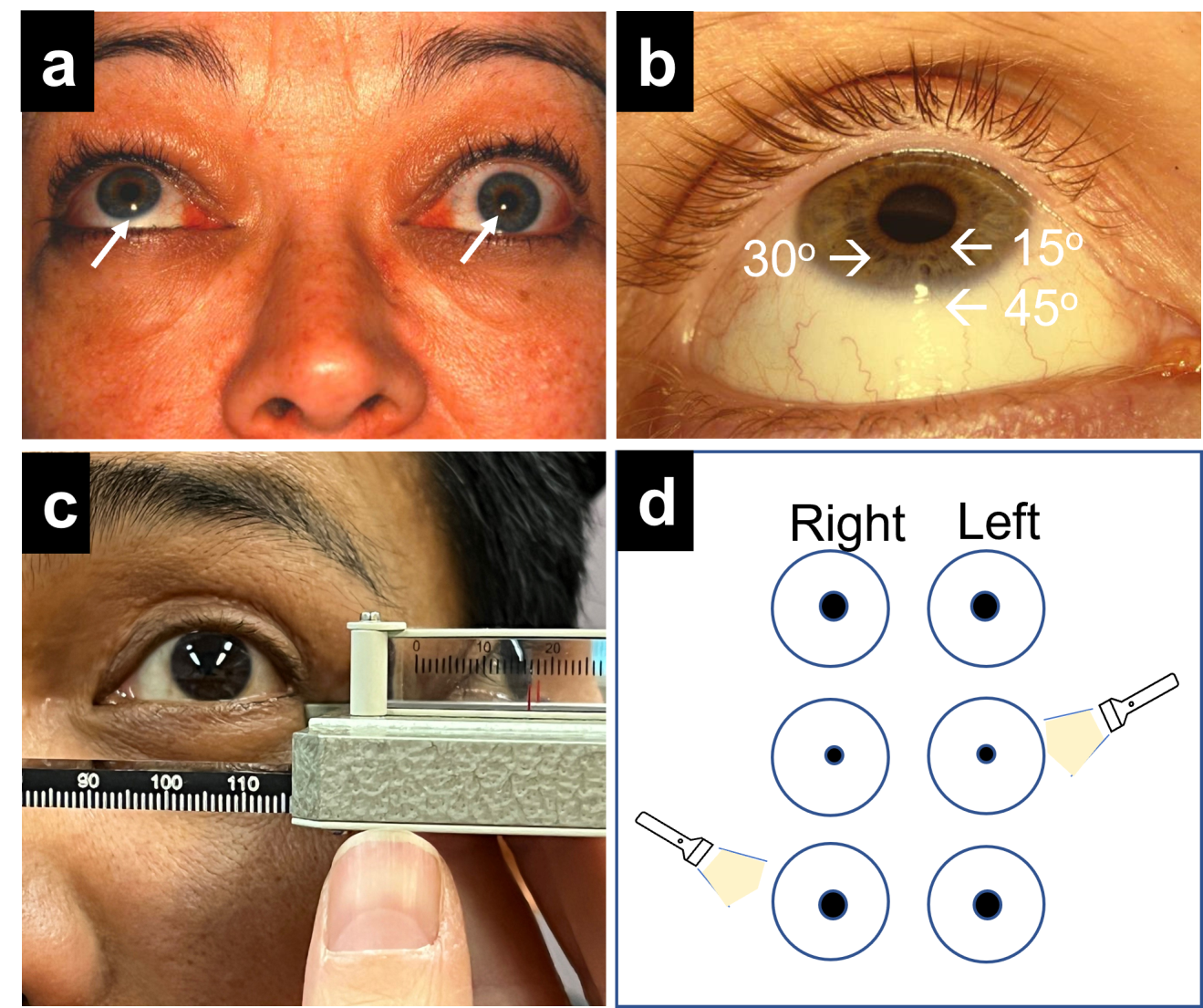


**Figure S1 Abbreviations:** TED: thyroid eye disease
