# Supplementary material for: Management of Thyroid Eye Disease: A Consensus Statement by the American Thyroid Association and the European Thyroid Association
Source: Thyroid. 2022 Dec 13;32(12):1439–70. doi: 10.1089/thy.2022.0251 (PMC9807259; doi:10.1089/thy.2022.0251)
Supplement: Supplemental data [file Supp_FigS2.docx]

**Figure S2: Surgical outcomes in TED** (Courtesy P Dolman)

**a, b**. Congestive orbitopathy: a**:** This patient had persistent periocular inflammatory signs with congestion and edema that was non-responsive to IVGC. She had no progression in any of her severity parameters for over 6 months. Although her CAS scores were very high, she had inactive disease (false positive CAS). b**.** 2 Weeks following orbital decompression, her soft tissue inflammatory signs had markedly improved (CAS reduced to zero) from reduction in venous congestion rather than disease inactivation.

**c:** Right asymmetric proptosis from fat expansion with lid retraction.

**d.** Improved symmetry following right orbital decompression for disfiguring proptosis along with upper eyelid lowering and lower eyelid elevation.

**e, f:** Quiescent orbitopathy with misaligned eyes, strabismus, and retracted eyelids, before and after ocular alignment and upper eyelid lowering surgery.

**g, h:** Isolated right upper eyelid retraction before and after levator recession surgery from a posterior approach

**i, j:** Bilateral upper lid retraction and upper lid and sub-brow fat expansion before and after upper lid lowering from an anterior approach along with fat sculpting blepharoplasty.

**
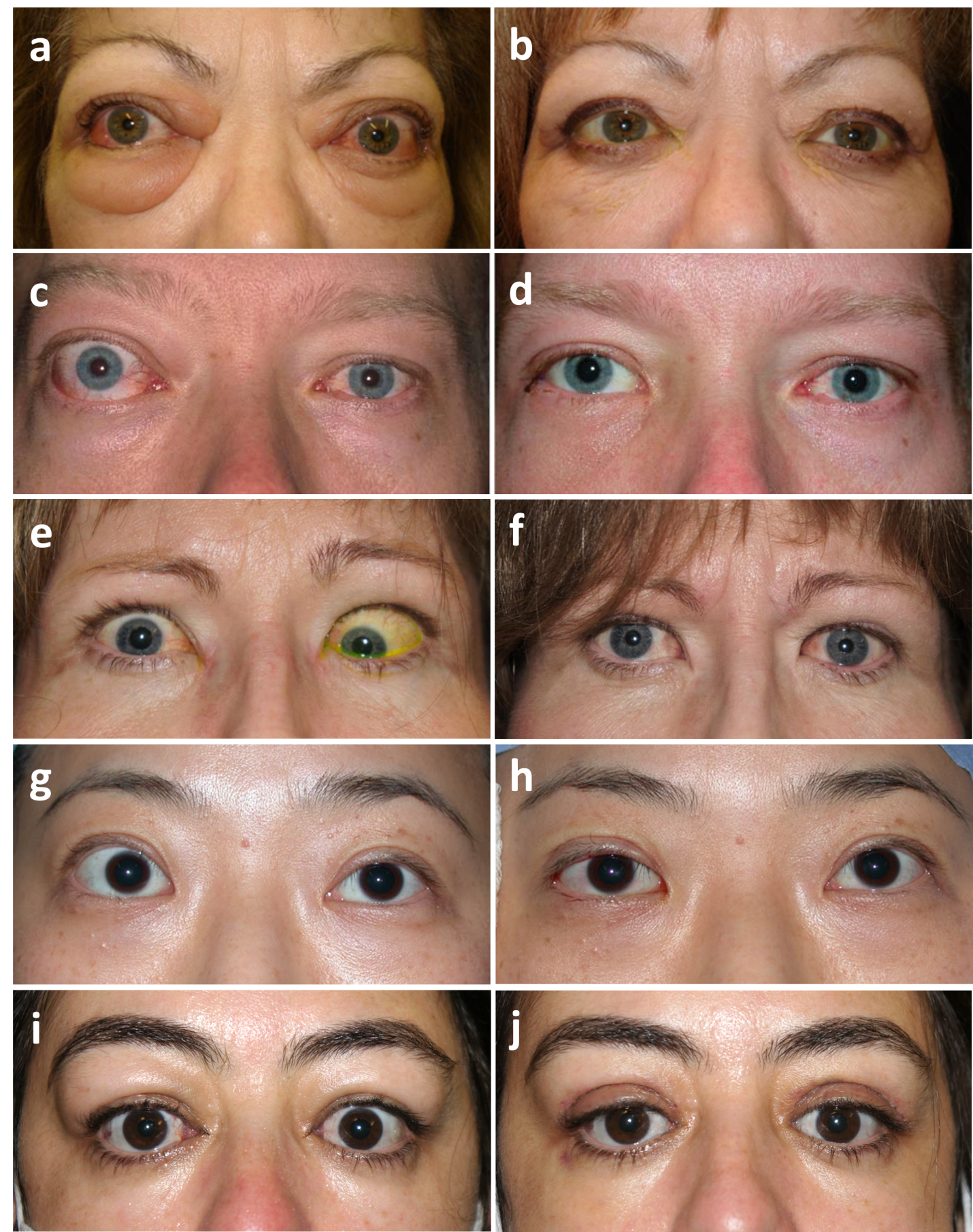
**

**Figure S2 Abbreviations:** CAS: clinical activity score; IVGC: intravenous glucocorticoids; TED: thyroid eye disease
