# Supplementary material for: Management of Thyroid Eye Disease: A Consensus Statement by the American Thyroid Association and the European Thyroid Association
Source: Thyroid. 2022 Dec 13;32(12):1439–70. doi: 10.1089/thy.2022.0251 (PMC9807259; doi:10.1089/thy.2022.0251)
Supplement: Supplemental data [file Suppl_TableS1.docx]

**Supplementary Table 1:** Conflict of Interest Disclosures ATA-ETA Thyroid Eye Disease Consensus Statement

| First Name | Last Name | Financial Disclosure Form? | FDF Date Received? | Disclosures Noted | Is this relationship  relevant? | Mitigation Measures |
| --- | --- | --- | --- | --- | --- | --- |
| Henry | Burch | Yes | 12/08/2020 | UpToDate | No |  |
| Petros | Perros | Yes | 11/03/2020 | IBSA Institut Biochimie | No |  |
| Tomasz | Bednarczuk | Yes | 11/04/2020 | Merck  Berlin-Chemie  Novartis  Ipsen  Roche diagnostics | No  No  No  No  No |  |
| David | Cooper | Yes | 3/31/2020 | None |  |  |
| Peter | Dolman | Yes | 12/16/2020 | Immunovant | No |  |
| Angela | Leung | Yes | 05/10/2021 | Vertice  Medscape  China Merck  IBSA Institut Biochimie | No  No  No  No |  |
| Ilse | Mombaerts. | Yes | 11/05/2020 | None |  |  |
| Mario | Salvi | Yes | 11/29/2020 | IBSA Institut Biochimie  Valenza-Bio  Roche - Institutional Funding only  Horizon- institutional support only | No  No  No  Yes | Recusal from discussion of teprotumumab, institutional support was considered non-exclusionary. |
| Marius | Stan | Yes | 10/16/2020 | Horizon- institutional support only  Immunovant  Siemens Healthineers  Tolmar | Yes  No  No  No | Recusal from discussion of teprotumumab; institutional support was considered non-exclusionary. |
